# Structure of UreG/UreF/UreH Complex Reveals How Urease Accessory Proteins Facilitate Maturation of *Helicobacter pylori* Urease

**DOI:** 10.1371/journal.pbio.1001678

**Published:** 2013-10-08

**Authors:** Yu Hang Fong, Ho Chun Wong, Man Hon Yuen, Pak Ho Lau, Yu Wai Chen, Kam-Bo Wong

**Affiliations:** 1 School of Life Sciences, Center for Protein Science and Crystallography, The Chinese University of Hong Kong, Hong Kong, China; 2 King's College London, Randall Division of Cell and Molecular Biophysics, London, United Kingdom; Brandeis University, United States of America

## Abstract

Urease is a metalloenzyme essential for the survival of *Helicobacter pylori* in acidic gastric environment. Maturation of urease involves carbamylation of Lys219 and insertion of two nickel ions at its active site. This process requires GTP hydrolysis and the formation of a preactivation complex consisting of apo-urease and urease accessory proteins UreF, UreH, and UreG. UreF and UreH form a complex to recruit UreG, which is a SIMIBI class GTPase, to the preactivation complex. We report here the crystal structure of the UreG/UreF/UreH complex, which illustrates how UreF and UreH facilitate dimerization of UreG, and assembles its metal binding site by juxtaposing two invariant Cys66-Pro67-His68 metal binding motif at the interface to form the (UreG/UreF/UreH)_2_ complex. Interaction studies revealed that addition of nickel and GTP to the UreG/UreF/UreH complex releases a UreG dimer that binds a nickel ion at the dimeric interface. Substitution of Cys66 and His68 with alanine abolishes the formation of the nickel-charged UreG dimer. This nickel-charged UreG dimer can activate urease *in vitro* in the presence of the UreF/UreH complex. Static light scattering and atomic absorption spectroscopy measurements demonstrated that the nickel-charged UreG dimer, upon GTP hydrolysis, reverts to its monomeric form and releases nickel to urease. Based on our results, we propose a mechanism on how urease accessory proteins facilitate maturation of urease.

## Abstract 2

Structural and biochemical study of urease accessory protein complex provides mechanistic insights into the delivery of nickel to metalloenzyme urease, an enzyme enabling the survival of *Helicobacter pylori* in the human stomach.

## Author Summary

Catalytic activities of many important enzymes depend upon metal cofactors. Ensuring each enzyme acquires the proper type of metal cofactor is essential to life. One such example is urease, which is a nickel containing metalloenzyme catalyzing the hydrolysis of urea to ammonia. The survival of *Helicobacter pylori*, a stomach ulcer–causing pathogen, in the human stomach depends on the ammonia released to neutralize gastric acid. In this study, we revealed the detail mechanism of how urease accessory proteins UreF, UreH, and UreG cooperate to couple GTP hydrolysis to deliver nickel to urease. UreF/UreH complex interacts with two molecules of GTPase UreG and assembles a metal binding site located at the interface between two UreG molecules. Nickel can induce GTP-dependent dimerization of UreG. This nickel-carrying UreG dimer together with UreF, UreH, and urease assemble into a protein complex. Upon stimulation of UreG GTPase activity by bicarbonate, UreG hydrolyses GTP and releases nickel into urease. Other nickel-delivering NTPases share similar properties with UreG; therefore, the nickel delivery mechanism described here is likely universally shared among these proteins.

## Introduction

More than a quarter of the cellular proteins are metalloproteins [1]. Homeostatic regulation on the incorporation of specific metal ions to these metalloproteins is essential to life. Metal ions that occupy the top of the Irving-Williams series [2], such as nickel, copper, and zinc, form stable complexes with proteins. Therefore, these metals must be tightly regulated in the cell to be kept out of other proteins that require less competitive metals to function. Specificity of metal incorporation into metalloprotein is conferred by specific protein–protein interactions with metallochaperones. Urease is a nickel containing metalloenzyme that hydrolyses urea into ammonia. It is a bacterial virulence factor that enables the survival of bacteria through the use of urea as the sole nitrogen source under nitrition limiting conditions [3]. Moreover, its activity enables *Helicobacter pylori* to survive under strongly acidic conditions of the human stomach by neutralizing gastric acid with the ammonia released [4]. Maturation of urease, a process that involves the delivery of two nickel ions into the carbamylated active site of the apo-enzyme, offers a paradigm to study metallochaperone-driven incorporation of metal into metalloenzyme.

Four urease accessory proteins—UreE, UreF, UreG, and UreH (UreH is an ortholog of UreD found in other species)—participate in the regulation of nickel delivery and urease maturation [5]. These genes were first identified in *Klebsiella aerogenes* using deletion and complementation approaches [6] and their homologs were later identified in *H. pylori* in a similar fashion [7]. *In vitro* pull-down assay showed that apo-urease forms a series of complexes with *K. aerogenes* urease accessory proteins—namely, UreD/urease [8], UreF/UreD/urease [9], and UreG/UreF/UreD/urease [10]. The interactions between urease and its accessory proteins were further supported by yeast-two-hybrid [11]–[13] and tandem affinity purification. It was hypothesized that the UreG/UreF/UreH complex is responsible for delivery of nickel ions into the urease active site and the formation of the preactivation complex is a critical step involved in urease maturation. Truncation study on *K. aerogenes* UreE–UreF fusion protein demonstrated that the C-terminal region of UreF is essential for interaction with other urease accessory proteins [14]. We have recently solved the crystal structure of UreF/UreH complex and demonstrated that UreH induces conformational changes in UreF to facilitate the recruitment of UreG [15]. Substitutions on the conserved residues at the C-terminal tail of UreF disrupt the formation of UreG/UreF/UreH complex [15]. Failure to recruit UreG to form UreG/UreF/UreH complex also resulted in abolishment of urease maturation [15],[16].

UreG is a SIMIBI (after signal recognition particle, MinD, and BioD) class GTPase involved in the regulation of nickel delivery [17]. Biological functions of SIMIBI GTPases are frequently regulated by dimerization [18]. Its GTPase activity is essential for urease maturation as either the substitution of GTPase P-loop motif or the use of nonhydrolyzable GTP analog during urease activation resulted in inactive urease [10],[19],[20]. UreG contains an invariant metal binding Cys-Pro-His motif [21],[22], substitution of which abolishes urease maturation [22]. It has been shown that UreG can interact with UreE, which was hypothesized as a nickel carrier that supplies nickel to urease during the maturation process [23]–[25].

How urease accessory proteins facilitate maturation of urease is not well understood. It is not known how GTP hydrolysis of UreG is coupled to urease activation, and why the recruitment of UreG to the urease preactivation complex is essential for urease maturation. Here, we report the structure of the UreG/UreF/UreH complex. Together with our biochemical study, we have revealed how urease accessory proteins couple GTP hydrolysis to nickel delivery, which may provide a paradigm for other metal-delivering NTPases.

## Results

### Crystal Structure of the UreG/UreF/UreH Complex

**Figure 1:**
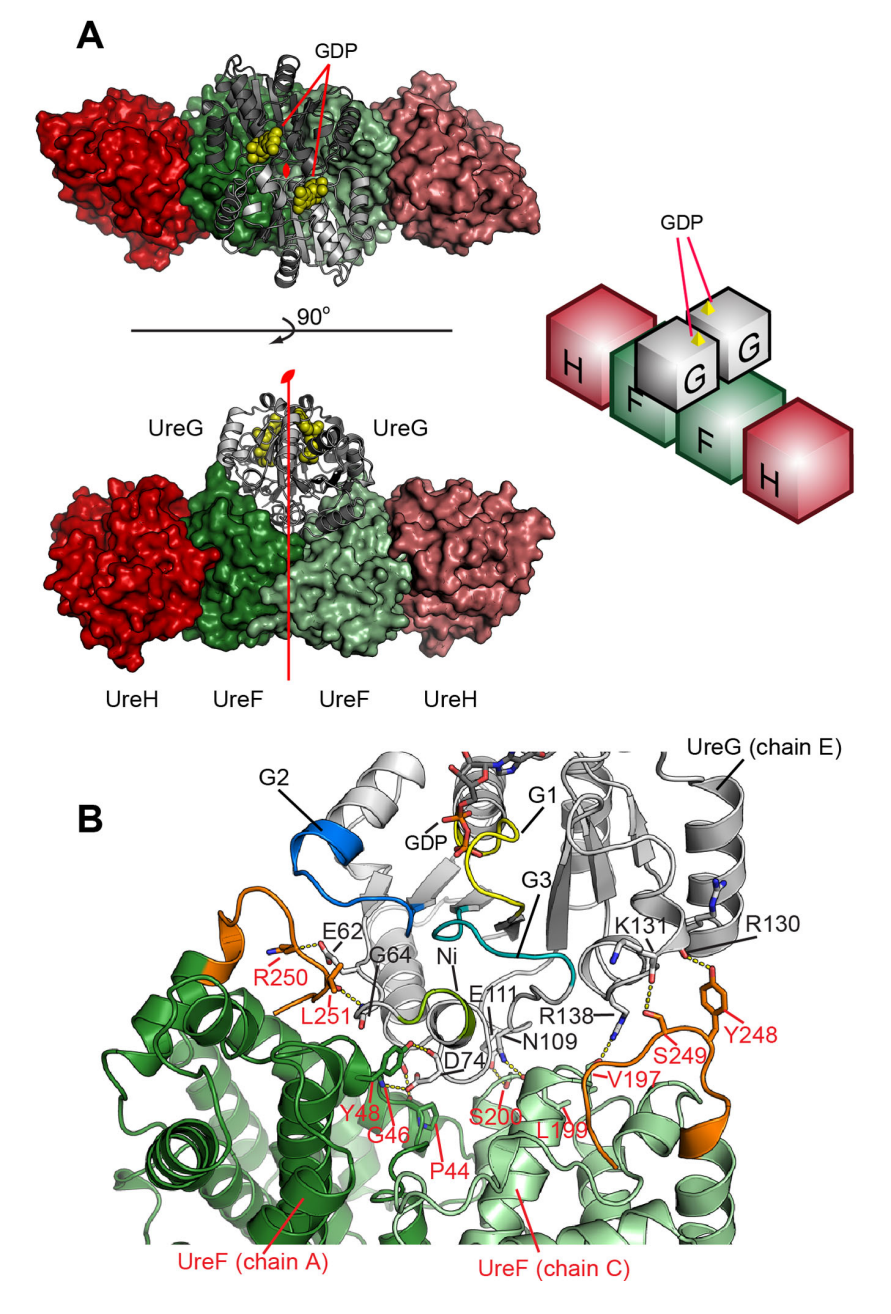
Structure of the UreG/UreF/UreH complex. (A) Structure of the UreG/UreF/UreH complex is shown on the left in two different orientations. UreF, UreH, and UreG are colored in shades of green, red, and grey, respectively. GDP ligands are shown as yellow space filling model. A schematic diagram of the relative positions of UreF, UreH, and UreG is shown on the right. The complex is a dimer of heterotrimers, with each pair of protomers related by a 2-fold symmetry. The dyad axis is shown. (B) Details of interactions between UreG and UreF. Only one UreG protomer (chain E) is shown for clarity. UreF protomers are colored in shades of green, and UreG is colored in white, with GTPase motifs (G1–G3) and metal binding motif (Ni) highlighted using the color scheme in Figure 2A. Residues involved in interaction between UreG (labeled in black) and UreF (labeled in red) are shown as sticks. A summary of the interactions is provided in Table S2. Note that each UreG protomer interacts with the F-tail loop of both UreF protomers (colored in orange).

**Figure 2:**
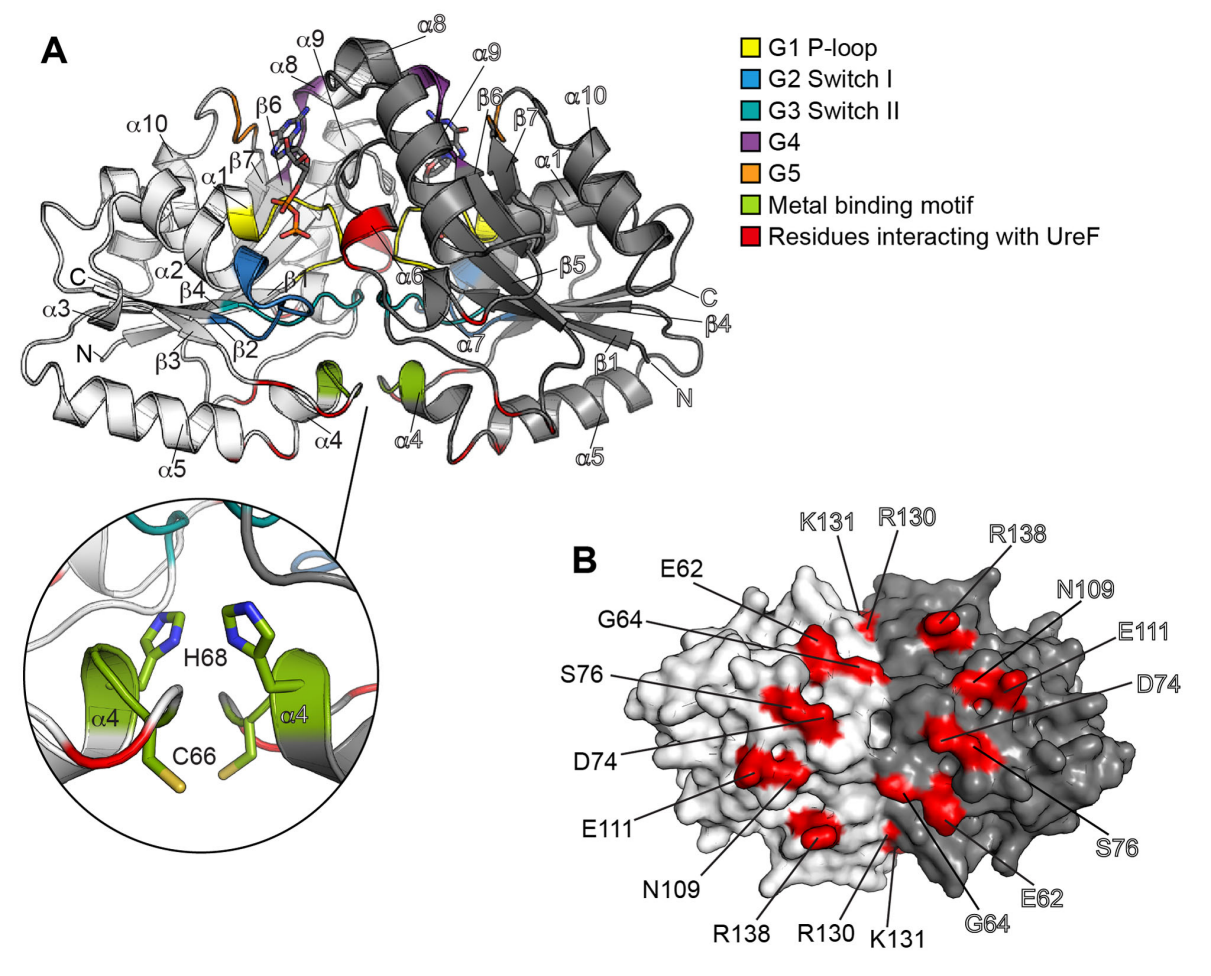
Structure of UreG. (A) Two UreG protomers are colored in white and grey, respectively, with secondary structural elements labeled. GTPase structural motifs (G1–G5), metal binding motif, and residues involved in interaction with UreF are colored as indicated. The inset shows the details of the metal binding site located at the interface between two UreG protomers, with residues involved in nickel chelation shown in sticks. (B) UreG is shown in surface representation and rotated horizontally by 90° relative to the orientation in (A). Surface of UreG residues involved in interaction with UreF are labeled and highlighted in red.

To gain insight into the urease maturation process, we determined the structure of the UreG/UreF/UreH complex in its GDP-bound state to 2.35 Å resolution using X-ray crystallography (Table S1). Coordinates of the structure have been deposited in the Protein Data Bank (PDB Code: 4HI0; the coordinates were communicated to Prof. R. P. Hausinger prior to publication and our structure was mentioned in a recent review article by Hausinger et al. [26]). The asymmetric unit contains two copies of each of UreF, UreH, and UreG, related by 2-fold symmetry, forming a dimer of heterotrimers (Figure 1A). UreF and UreH in the UreG/UreF/UreH complex have essentially the same conformation as previously reported in the UreF/UreH complex (Figure S1, Cα r.m.s.d. 0.742 Å). The UreG/UreF/UreH complex structure represents the first known structure of UreG (Figure 2A). The topology of UreG is characteristic of SIMIBI class GTPases containing the canonical G1 to G5 motif for guanine nucleotide recognition (Figure S2). Two GDP ligands are sandwiched between two UreG protomers (Figure 1A). UreG contains an invariant Cys66-Pro67-His68 metal binding motif (Figure S3) [27], substitution of which abolishes the metal binding properties of UreG [22],[27]. This metal binding motif is found at the dimer interface between two UreG protomers (Figure 2A). Cys66 and His68 from each protomer are arranged in a symmetric formation that could potentially bind a metal ion at the interface (Figure 2A).

The crystal structure also showed that UreG interacts strictly with UreF only, leading to an overall structure resembling that of an inverted-T shape (Figure 1A). Each UreG protomer makes extensive contacts with both protomers of UreF. The contact surface area between one UreG protomer (chain E) and UreF chain A is 1,664 Å^2^ and with chain C is 1,004 Å^2^, respectively. The interaction between UreG and UreF is mostly mediated via specific hydrogen bonds and salt bridges (Table S2), with the interacting residues distributed evenly in a ring-shaped pattern on the UreG dimer surface contacting UreF (Figure 2B). These interacting residues are positioned in close proximity to the GTPase switch I and II (G2 and G3) motifs and the metal binding motifs of UreG (Figures 1B and 2A). Moreover, each UreG protomer is in intimate contact with the residues on the F-tail loops of both protomers of UreF (Figure 1B). This observation is consistent with previous structural and mutagenesis analyses, which suggest that binding of UreH induces the ordering of the F-tail loop structure required for the recruitment of UreG [15],[16].

### Homodimerization of UreF/UreH Complex Is Essential for the Recruitment of UreG

**Figure 3:**
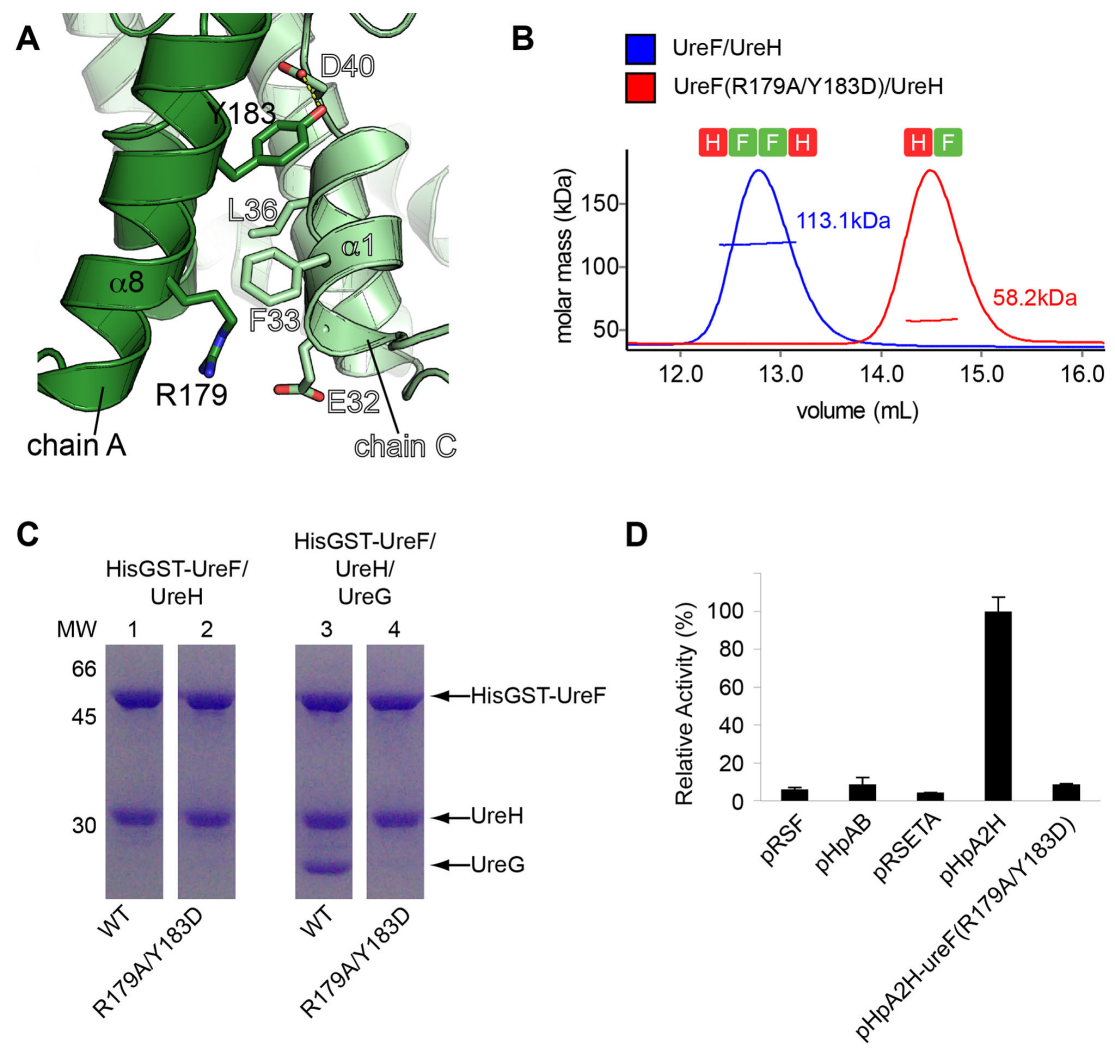
Homodimerization of UreF/UreH is essential to urease maturation. (A) Design of mutant. R179A substitution on UreF removes the electrostatic interaction between Arg179 and Glu32. Y183D substitution removes the hydrogen bond between Tyr183 and Asp40, and disfavors the packing of helix α8 against the hydrophobic residues of helix α1. (B) Substitution of R179A/Y183D on UreF disrupts the homodimerization of the UreF/UreH complex. Molecular weights of wild-type UreF/UreH complex and its variant, measured using SEC/SLS, were 113.1±0.7 and 58.2±0.2 kDa, which are consistent with the expected molecular weights of (UreF/UreH)_2_ and (UreF/UreH), respectively. (C) UreF(R179A/Y183D) variant cannot recruit UreG. GST pull-down assay showed that both wild-type (lane 1) and the UreF(R179A/Y183D) variant (lane 2) co-eluted with UreH, suggesting that the UreF variant retains the ability to form a complex with UreH. However, UreG co-eluted with wild-type HisGST-UreF (lane 3) but not with the R179A/Y183D UreF variant (lane 4), indicating the ability to recruit UreG is lost in the UreF(R179A/Y183D)/UreH variant. (D) The UreF(R179A/Y183D) variant cannot activate urease *in vivo*. The *in vivo* urease activity was carried out by transforming the pHpA2H plasmid containing the entire urease operon (*ureABIEFGH*) in a pRSETA vector into an *E. coli* host. The pHpAB plasmid contains the urease structural gene (*ureAB*) on a pRSFDuet vector and was used as a negative control. pHpA2H plasmid gave urease activity of 0.31±0.02 µmol NH_3_ mg^−1^ min^−1^. In contrast, pHpA2H plasmid containing *ureF*(R179A/Y183D) mutation gave urease activity of less than 10% of pHpA2H.

As each UreG protomer interacts with both protomers of UreF (Figure 1B), we hypothesized that dimerization of UreF/UreH to form the (UreF/UreH)_2_ complex is prerequisite to UreG recruitment. To test this hypothesis, we created a UreF variant (R179A/Y183D) (Figure 3A) that disrupted the homodimerization of UreF/UreH (Figure 3B). We showed that the substitutions resulted in a dimerization-deficient UreF(R179A/Y183D)/UreH complex that failed to recruit UreG (Figure 3C). Next, we test if the UreF variant can activate urease *in vivo* (Figure 3D). Whereas the wild-type pHpA2H plasmid gave an activity of 0.31±0.02 µmol NH_3_ mg^−1^ min^−1^, pHpA2H-*ureF*(R179A/Y183D) mutant plasmid gave activity similar to that of the negative control of pHpAB, containing only the urease structural genes (Figure 3D). Taken together, our results suggest that homodimerization of the UreF/UreH complex is essential for the recruitment of two copies of UreG to form the (UreG/UreF/UreH)_2_ complex and urease maturation.

### GTP and Nickel Induces Dissociation of UreG Dimer From the UreF/UreH Complex

**Figure 4:**
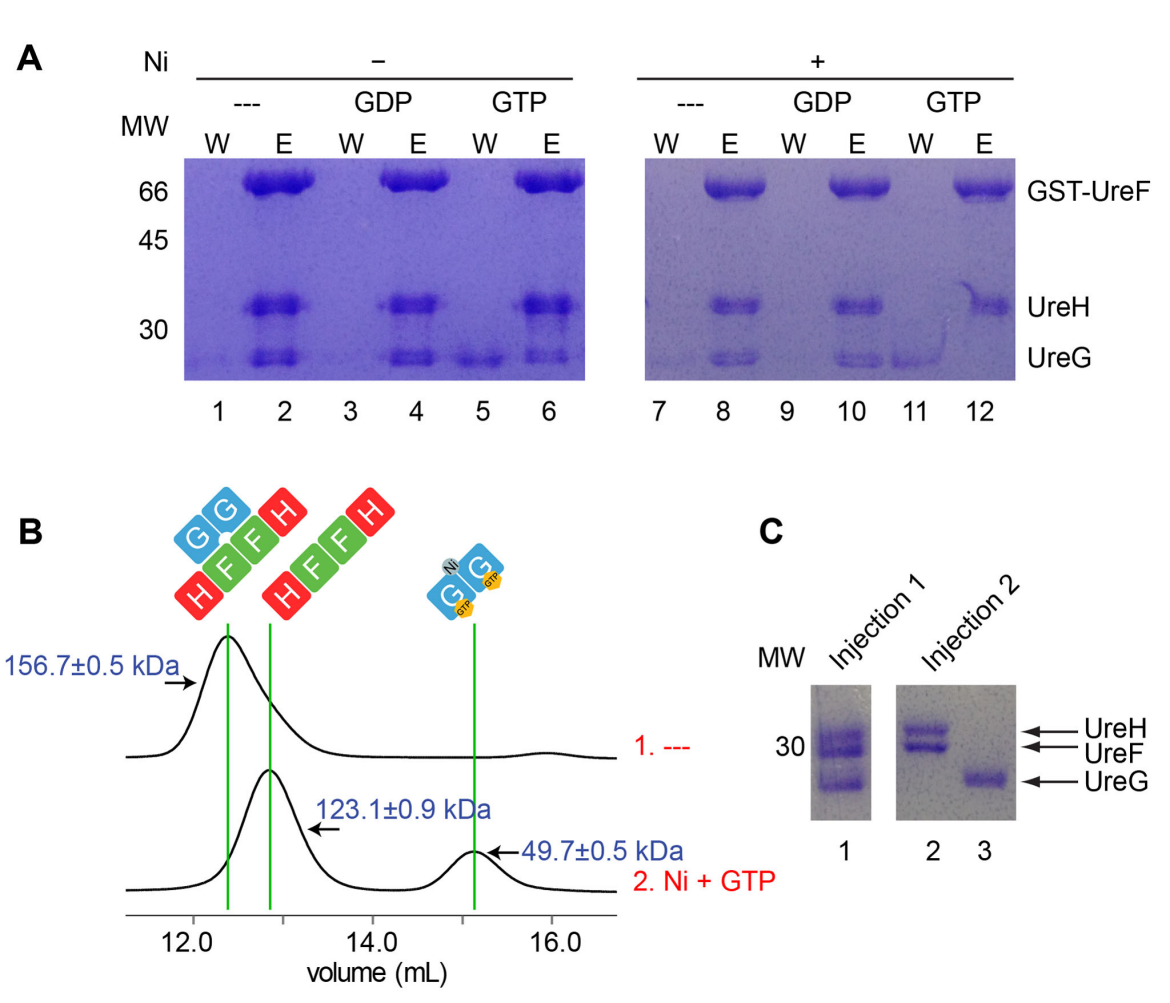
UreG dissociates from the UreF/UreH complex in the presence of nickel and GTP. (A) To test the effect of nickel and guanine nucleotide on the UreG/UreF/UreH complex, the GST-UreF/UreH/UreG complex was first immobilized on GST Spintrap columns. After washing with different combinations of 0.5 mM nickel and/or 1 mM GDP/GTP (lanes 1, 3, 5, 7, 9, and 11), proteins remained on the column were eluted with glutathione (lanes 2, 4, 6, 8, 10, and 12). The wash (W) and eluted (E) fractions were analyzed using SDS-PAGE. UreG partially dissociated from the UreG/UreF/UreH complex upon incubation with GTP (lanes 5 and 6) and completely dissociated from the complex upon incubation with GTP and nickel (lanes 11 and 12). (B and C) Analytical gel filtration profiles of the UreG/UreF/UreH complex with (injection 2) or without (injection 1) prior incubation with 0.5 mM nickel and 1 mM GTP (B). Molecular weights measured (in kDa) using static light scattering are indicated. Eluted protein peaks were analyzed using SDS-PAGE (C). The UreG/UreF/UreH complex eluted with a molecular weight of 156.7±0.5 kDa, which is consistent with that of a heterohexameric (UreG/UreF/UreH)_2_ complex (C, lane 1). In contrast, after incubation with nickel and GTP, the UreG/UreF/UreH complex eluted as two distinct peaks with molecular weights of 123.1±0.9 kDa and 49.7±0.5 kDa, respectively, which correspond to the molecular weights of (UreF/UreH)_2_ complex (C, lane 2) and UreG dimer (C, lane 3).

In an attempt to prepare the UreG/UreF/UreH complex in its GTP-bound state, we have accidentally discovered that UreG has a tendency to dissociate from the UreG/UreF/UreH complex upon addition of GTP. We performed GST pull-down experiments to test the interaction between UreG and UreF/UreH in the presence of GDP or GTP. Our results showed that UreG partially dissociated from the GST-UreF/UreH complex in the presence of GTP (Figure 4A, lanes 5 and 6). On the other hand, complete dissociation of UreG was observed upon addition of both GTP and nickel (Figure 4A, lanes 11 and 12). Similar observation was obtained when GTP and zinc was added (Figure S4, lanes 5 and 6). These observations suggest that GTP binding promotes dissociation of UreG from the UreF/UreH complex, the process of which is enhanced by addition of nickel or zinc.

That the addition of both GTP and nickel dissociated UreG from the UreF/UreH complex was further supported by size exclusion chromatography/static light scattering (SEC/SLS) analysis. The UreG/UreF/UreH complex was eluted as a single peak at 12.5 ml, with a molecular weight of 156.7±0.5 kDa, which is consistent with the expected molecular weight of a heterohexameric (UreG/UreF/UreH)_2_ complex (Figure 4B, injection 1). Addition of GTP and nickel ions to the UreG/UreF/UreH complex resulted in elution of two peaks at 12.9 ml and 15.1 ml with molecular weights consistent with that of the UreF/UreH complex and UreG dimer (Figure 4B, injection 2). SDS-PAGE analysis of the eluted fractions confirmed the identity of the proteins eluted in the corresponding peaks (Figure 4C).

### UreG Forms a Nickel-Charged Dimer in the Presence of Both GTP and Nickel

**Figure 5:**
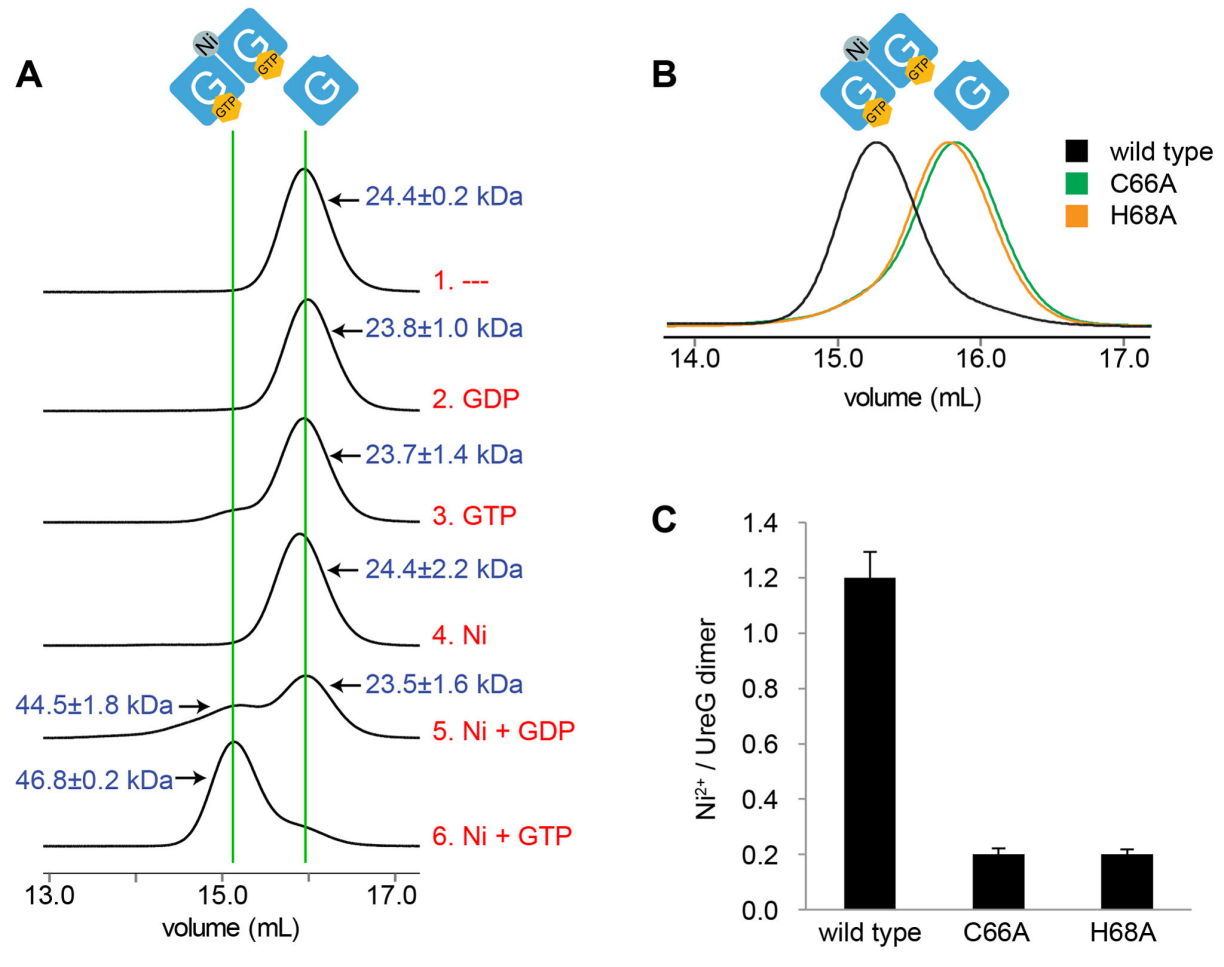
UreG dimerization is dependent on GTP and nickel ions. (A) We pre-incubated 40 µM UreG with 1 mM GTP/GDP and/or 0.5 mM nickel ions, and then injected them to Superdex S200 analytical gel filtration column pre-equilibrated with phosphate buffer saline. Molecular weights measured using static light scattering (in kDa) were indicated. UreG behaves as a dimer in the presence of nickel and GTP, while it remained mostly as monomers under other conditions. (B) Analytical gel filtration elution profiles of wild-type UreG and its variants in the presence of 1 mM GTP and 0.5 mM nickel ions. While wild-type UreG eluted as dimers in the presence of GTP and nickel ions, both UreG(C66A) and UreG(H68A) variants eluted as monomers. (C) Nickel content of UreG determined using atomic absorption spectroscopy. In the presence of nickel and GTP, wild-type UreG binds 1.2±0.1 nickel ions per UreG dimer, whereas significantly less nickel is bound to UreG(C66A) or UreG(H68A) variants.

The dissociation of UreG dimer from UreF/UreH complex upon the addition of GTP and nickel prompted us to systematically characterize the oligomerization state of UreG in the presence of GDP, GTP, and/or nickel ions. SEC/SLS analysis showed that regardless of the addition of guanine nucleotides, UreG eluted in a monomeric form (molecular weight ∼24 kDa) in the absence of nickel (Figure 5A, injections 1 to 3). In agreement with a previous study, we found that UreG remains a monomer in the presence of nickel (Figure 5A, injection 4) [21]. UreG showed some tendency to dimerize in the presence of nickel and GDP as indicated by the presence of a minor peak with molecular weight of 44.5±1.8 kDa (Figure 5A, injection 5). In the presence of both GTP and nickel ion, however, UreG eluted as a stable dimer with a molecular weight of 46.8±0.2 kDa (Figure 5A, injection 6). We further accessed the nickel content of UreG dimers by collecting the protein sample eluted from the SEC/SLS system and measuring its nickel content using atomic absorption spectroscopy (AAS). Our result shows that the protein sample contained 1.2±0.1 bound nickel per UreG dimer (Figure 5C). To demonstrate that the invariant Cys66-Pro67-His68 metal binding motif of UreG is responsible for its GTP-dependent dimerization and nickel binding properties, we repeated the above experiments using UreG(C66A) and UreG(H68A) variants. We found that while wild-type UreG dimerizes in the presence of GTP and nickel, both UreG variants remained as monomers (Figure 5B). Both UreG variants also have significantly reduced nickel chelating ability as compared to the wild-type UreG (Figure 5C). Thus, our data suggest that the UreG dimer binds a nickel ion with the invariant Cys66-Pro67-His68 motif at the dimeric interface.

### UreG Dimer Releases Nickel upon GTP Hydrolysis

As observed in the crystal structure of the UreG/UreF/UreH complex, the metal binding motif of UreG is situated at the interface between two UreG protomers (Figure 2A). Moreover, SEC/SLS analysis of UreG above suggested that the oligomerization state of UreG is related to its guanine nucleotide state (Figure 5A). These observations give rise to an attractive hypothesis that the transitioning of UreG between its monomeric and dimeric forms enables it to dynamically assemble and disassemble the metal binding site to deliver nickel to its target urease.

**Figure 6:**
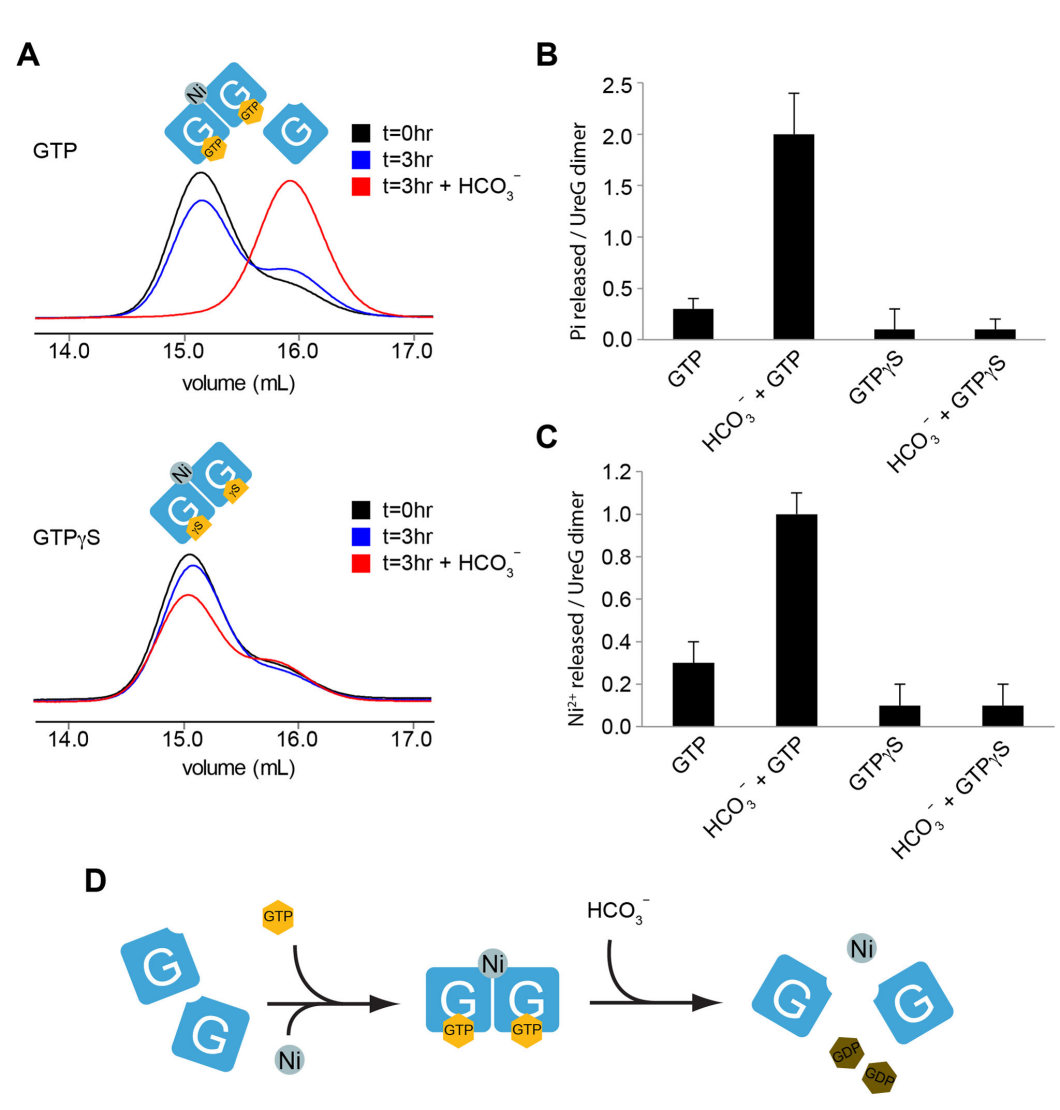
UreG dimer releases nickel upon GTP hydrolysis. (A) Nickel-charged UreG dimers return to a monomeric state upon GTP hydrolysis. Nickel-charged UreG dimers prepared using GTP (upper panel) or GTPγS (lower panel) were incubated for 3 h in the presence or absence of 100 mM bicarbonate ion. Analytical gel filtration profiles of UreG are shown. Most of the GTP-containing UreG dimers remained dimers after incubation, whereas most became monomers after incubation in the presence of bicarbonate. In contrast, GTPγS-containing UreG dimers remained dimers regardless of the presence of bicarbonate. (B and C) Phosphate and nickel released by UreG dimers in the presence and absence of bicarbonate were assessed by malachite green assay (B) and atomic absorption spectroscopy (C), respectively. Each nickel-charged UreG dimer released 2.0±0.4 phosphates and 1.0±0.1 nickel ions in the presence of bicarbonate. In comparison, only low levels of phosphate and nickel were released by UreG in the absence of bicarbonate or by UreG dimers prepared with GTPγS. (D) The nickel-charged UreG dimer releases its nickel upon stimulation of GTPase activity by bicarbonate.

We first tested if the nickel-charged UreG dimer can revert to monomer upon hydrolysis of bound GTP. Since bicarbonate is a known cofactor required for urease activation [28],[29], we speculated that bicarbonate stimulates GTP hydrolysis by UreG. We found that after incubation in the absence of bicarbonate at 37°C for 3 h, UreG mostly remained in the dimeric state (Figure 6A, upper panel). In contrast, all the UreG became monomeric in the presence of bicarbonate (Figure 6A, upper panel). We then measured the phosphate released during the incubation period using a malachite green-based assay. We found that each nickel-charged UreG dimer, when stimulated by bicarbonate, released 2.0±0.4 phosphate (Figure 6B), whereas only a negligible amount of phosphate was released when GTP and nickel are incubated with bicarbonate in the absence of UreG (Figure S5). In comparison, only 0.3±0.1 phosphate was released in the absence of bicarbonate (Figure 6B). When the experiment was repeated using UreG dimers prepared with GTPγS, which is a nonhydrolyzable analog of GTP, we found that UreG remained mostly in its dimeric form regardless of the presence of bicarbonate (Figure 6A, lower panel). Our results suggest that GTP hydrolysis stimulated by bicarbonate dissociates UreG dimer into monomer. In addition, we found that neither acetate, formate, nor sulfate is capable of stimulating phosphate release from UreG dimer (Figure S6). Therefore, stimulation of UreG GTPase activity appears to be specific to bicarbonate.

We then tested whether the bound nickel of UreG dimer is released upon GTP hydrolysis. We compared the nickel content of the dimeric and monomeric forms of UreG using AAS. Our results showed that after 3 h incubation in the presence of bicarbonate, each GTP-containing UreG dimer released 1.0±0.1 nickel ion. In comparison, less than 0.10 nickel was released from UreG dimer containing GTPγS (Figure 6C). Taken together, monomeric UreG dimerizes in the presence of both GTP and nickel ions. Stimulated by bicarbonate, UreG hydrolyzes GTP to GDP and returns to its monomeric form while releasing nickel ions in the process (Figure 6D).

### Nickel, But Not Zinc, Induces GTP-Dependent Dimerization of UreG

To test the metal specificity of GTP-dependent dimerization of UreG, we compared the dimerization behavior of UreG with nickel or zinc using SEC/SLS. UreG was mixed with nickel or zinc sulfate in the absence/presence of guanine nucleotides (Figure S7A). The protein sample was then loaded to a gel filtration column and eluted with a buffer without metal ions. Our results showed that UreG only formed a stable dimer with the addition of both nickel and GTP in the protein sample.

It was reported that zinc can induce dimerization of UreG [21],[30]. However, in their experiments, they included 10 µM zinc in the gel filtration buffer. Consistent with their finding, we found that UreG dimerized irrespective to the guanine nucleotide state when zinc was included in the gel filtration buffer (Figure S7B), but remained monomeric when the metal was absent from the buffer (Figure S7A). These findings suggest that zinc-induced UreG dimer is stable only when a constant amount of free zinc ion is in solution. In contrast, UreG was able to maintain a stable dimeric form with GTP even when nickel was not present in the gel filtration buffer (Figure S7A). Given the limited pool of free metal ions in the cytosol under cellular conditions, we deem that GTP-dependent dimerization of UreG is specific for nickel.

### Nickel-Charged UreG Dimer Can Activate Urease in the Presence of UreF/UreH Complex

**Figure 7:**
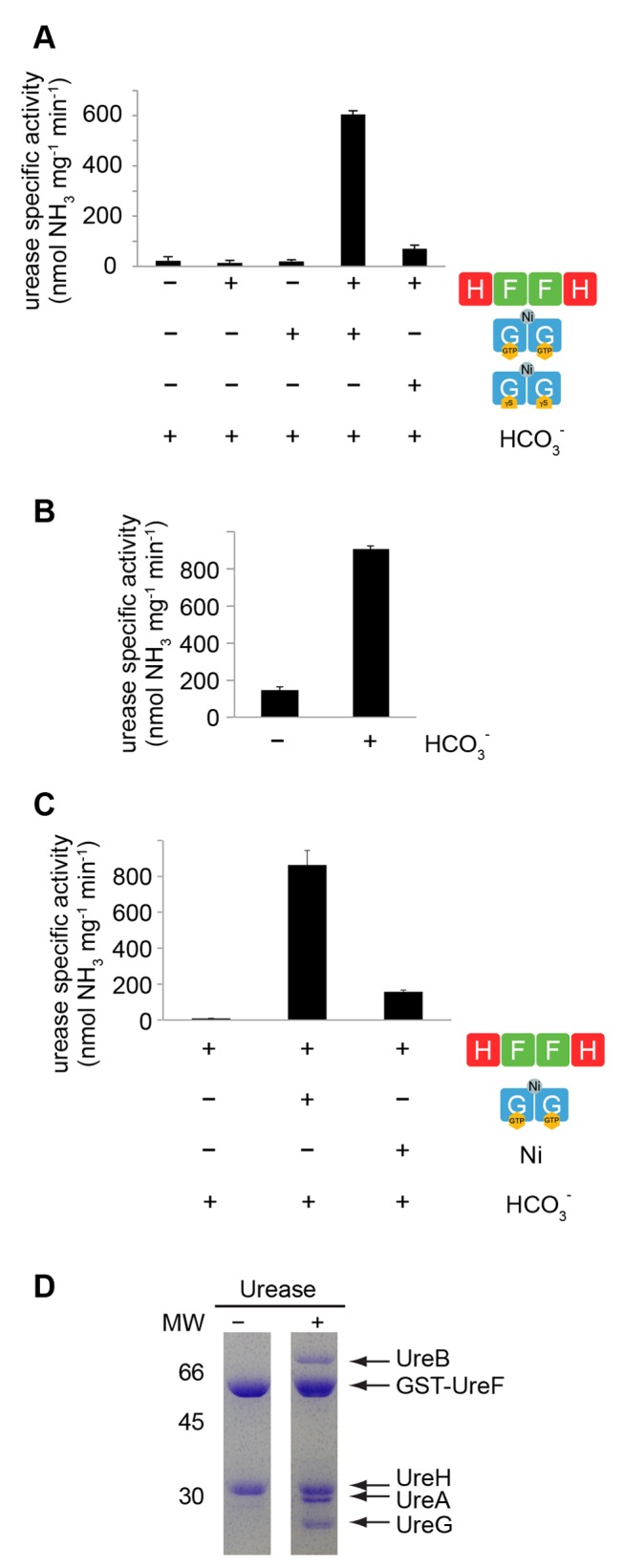
Nickel-charged UreG dimer can activate urease *in vitro* in the presence of UreF/UreH. (A) *In vitro* urease assay was performed by incubating apo-urease with purified samples of UreF/UreH, bicarbonate ion and nickel-charged UreG dimers containing either GTP or GTPγS. In the presence of UreF/UreH, the urease was activated, with a specific activity of 605±14 nmol NH_3_ mg urease^−1^ min^−1^, by the nickel-charged UreG dimer containing GTP, but not by the UreG dimer containing GTPγS. For the purpose of comparison, *H. pylori* urease activity has been reported to be in the range of ∼10–1,000 µmol NH_3_ mg^−1^ min^−1^ [23],[37],[58]. (B) *In vitro* urease assay was performed by incubating apo-urease, UreF/UreH, and nickel-charged UreG dimers in the presence or absence of 100 mM bicarbonate. (C) *In vitro* urease assay was performed by incubating apo-urease and UreF/UreH with bicarbonate ion and 20 µM of nickel-charged UreG dimers or 20 µM free nickel. (D) GST-UreF/UreH or GST-UreF/UreH/urease was first immobilized on a 5 ml GSTrap column. Purified GTP/nickel-charged UreG dimer was loaded onto the column and incubated for 15 min. After washing to remove excess UreG, protein was eluted with glutathione and analyzed using SDS-PAGE. Nickel-charged UreG dimer does not interact with the GST-UreF/UreH complex but interacts with the GST-UreF/UreH/urease complex.

Considering both structural and biochemical data, we showed that dimerization of the UreF/UreH complex provides a platform to recruit two copies of UreG in a GDP-bound state to form a heterohexameric complex (UreG/UreF/UreH)_2_. Addition of nickel and GTP dissociates the complex into the (UreF/UreH)_2_ complex and a nickel-charged UreG dimer (Figure 4). We then questioned whether the nickel-charged UreG dimer is biologically active and capable of activating urease. To this end, we performed an *in vitro* urease activation assay using purified proteins, without external sources of nickel or GTP other than those from the UreG dimers. We found that the nickel-charged UreG dimer can activate urease only in the presence of the UreF/UreH complex (Figure 7A). In contrast, without the addition of UreF/UreH, the nickel-charged UreG dimer alone was not capable of urease activation (Figure 7A). Moreover, only GTP-bound UreG dimers could activate urease but not those with GTPγS (Figure 7A), suggesting that GTP hydrolysis is a requirement for urease activation. Consistent with the observation that bicarbonate can stimulate GTPase activity of UreG, we also found that urease can be activated only in the presence of bicarbonate (Figure 7B). Since bicarbonate can also stimulate the release of nickel from the UreG dimer (Figure 6), we tested if free nickel at a concentration equivalent to that of UreG dimer (20 µM) can activate urease. Our results showed that the addition of nickel resulted in only ∼18% urease activation in the absence of UreG (Figure 7C). This observation suggests that the bound nickel in the UreG dimer is important in urease activation, and it is likely that GTP hydrolysis of UreG and nickel release have to couple with the UreF/UreH/urease complex to achieve efficient urease activation.

Nickel-charged UreG dimer can activate urease *in vitro* in the presence of UreF/UreH (Figure 7A), suggesting nickel ions were transferred from the UreG dimer to urease. We next tested whether the nickel-charged UreG dimer can interact with the UreF/UreH/urease complex. Pull-down assay showed that the nickel-charged UreG dimer does not interact with the GST-UreF/UreH complex (Figure 7D). This indicates that the nickel-charged UreG dimer does not reassociate with the UreF/UreH complex. Interestingly, we found that the nickel-charged UreG dimer can interact with the GST-UreF/UreH/urease complex (Figure 7D). Presumably, conformational changes resulting from the formation of the UreF/UreH/urease complex are necessary to accommodate the UreG dimer. Nevertheless, our result implies that nickel is transferred from UreG to urease through the complex formation with UreF/UreH and urease.

## Discussion

Urease is a metalloenzyme whose catalytic activity is crucially dependent on the placement of the proper metal ion in the active site. This is achieved *in vivo* through the use of four urease accessory proteins in a GTP-hydrolysis-dependent process [10]. It is well known that these urease accessory proteins form a series of complexes with urease [5], leading to the formation of the urease preactivation complex consisting of UreF, UreH, UreG, and urease. Crystal structures of the UreF and UreF/UreH complex provided a structural rationale for the sequence of assembling components in the preactivation complex [15],[31]. Using chemical cross-linking, mutagenesis, and SAXS experiments, it has been shown that UreF/UreH binding induces conformational changes on urease [32],[33]. UreF has been suggested to increase the fidelity of nickel incorporation in urease [16]. However, less is understood on how nickel is inserted into urease.

In this study, we have determined the crystal structure of the UreG/UreF/UreH complex. Static light scattering measurements established that while apo-UreG is monomeric in solution (Figure 5A), the complex exists in a dimeric (UreG/UreF/UreH)_2_ formation similar to that observed in the crystal structure (Figure 4B). Together, the structure shows that (UreF/UreH)_2_ provides a scaffold for UreG dimerization. In support of this, it has been previously observed that UreF variants with substitution of conserved residues at the UreF-UreG interface abolished both recruitment of UreG and maturation of urease [15],[16]. Moreover, UreF/UreH dimerization is essential to urease maturation, as suggested by our mutagenesis study showing that UreF with substitutions to disrupt the homodimerization of UreF/UreH complex also failed to recruit UreG and abolishes urease maturation (Figure 3). Taken together, we conclude that UreF/UreH-assisted dimerization of UreG is a prerequisite for the production of active urease.

Our structure further indicates that dimerization of UreG brings together the invariant Cys-Pro-His metal binding motifs on each UreG protomer, suggesting that nickel is chelated at the dimeric interface of UreG (Figure 2A). Next, we showed that nickel can induce GTP-dependent dimerization of UreG (Figure 5A) and that a stable nickel-charged UreG dimer can be purified. The role of the Cys-Pro-His motif in binding metal was confirmed by the observation that UreG variants with either C66A or H68A substitutions abolished nickel-induced GTP-dependent dimerization of UreG (Figure 5B). Furthermore, equivalent substitutions on *K. aerogenes* UreG abolished urease maturation [22]. Together, this suggests UreG dimerization results in the chelation of a nickel ion that is essential to urease maturation. Further experimentation with the UreG dimer showed that nickel is released from this dimer upon GTP hydrolysis (Figure 6B and 6C), which accounts for the previously shown requirement of GTP hydrolysis for urease activation [10].

**Figure 8:**
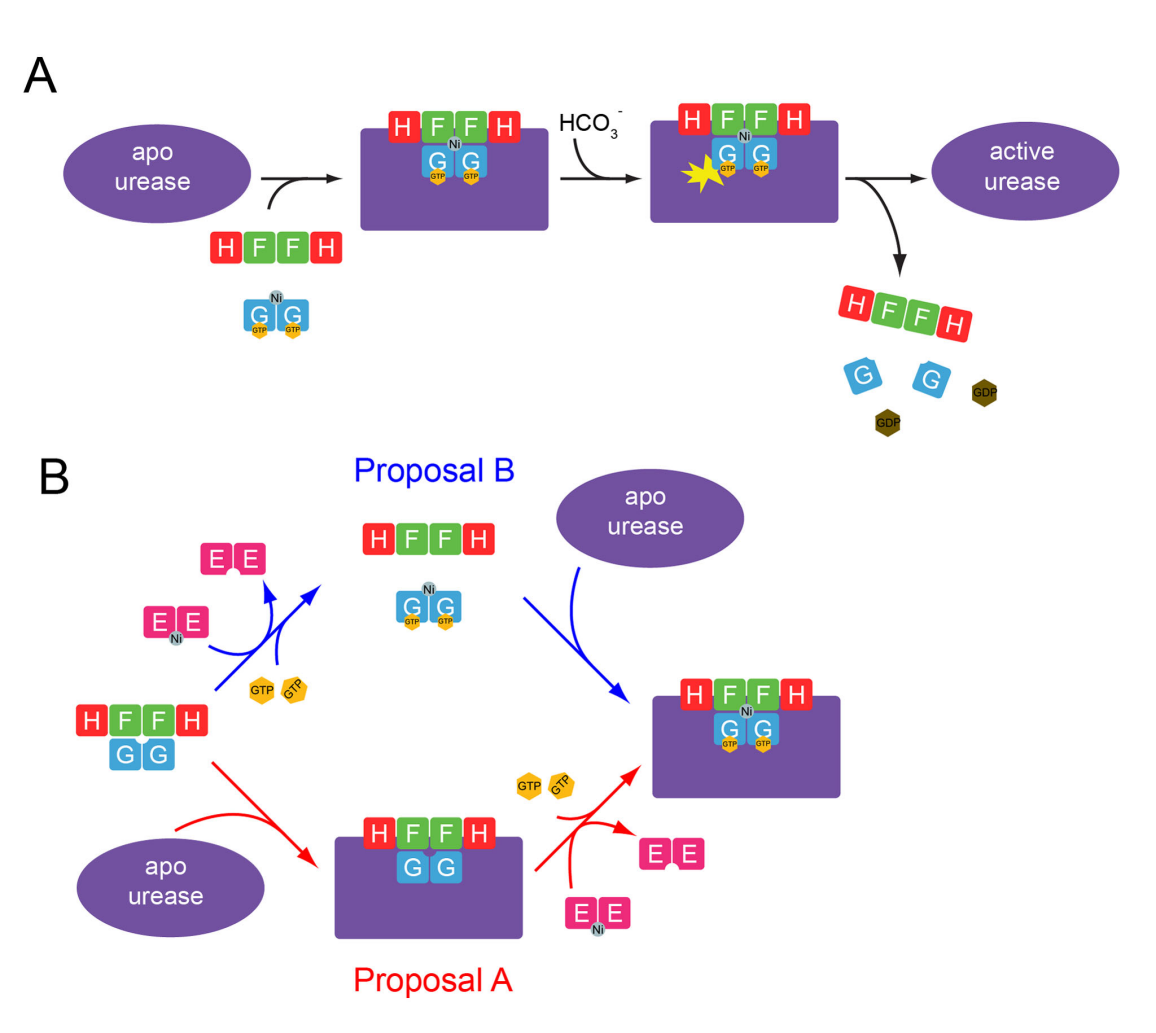
The proposed model for urease maturation. (A) The nickel-charged UreG dimer can form a complex with UreF/UreH and urease. It is anticipated that complex formation induces conformational changes in urease. After formation of the preactivation complex, bicarbonate stimulates the GTPase activity of UreG, leading to the delivery of nickel ion into the urease active site. (B) There are two possible mechanisms for how the preactivation complex (UreG/UreF/UreH/urease) receives its nickel from UreE. In proposal A (red arrows), the UreG/UreF/UreH first forms a complex with urease. UreE may interact with and transfer nickel to the complex as proposed by Hausinger et al. [26]. In proposal B (blue arrows), binding of GTP promotes dissociation of UreG from the UreG/UreF/UreH complex. The GTP-bound UreG then receives its nickel from UreE, giving rise to the GTP/nickel-charged UreG dimer, which can activate UreF/UreH-bound apo-urease in a GTP-hydrolysis-dependent process.

Our results also provide insights into how UreG couples GTP hydrolysis to the delivery of nickel to urease [10]. We showed that the nickel-charged UreG dimer can form a complex with UreF/UreH and urease, which upon stimulation of GTPase activity with bicarbonate resulted in activated urease (Figure 7). How the UreG dimer interacts with UreF/UreH/urease is not known. Since the UreG dimer only interacts with the UreF/UreH/urease complex but not UreF/UreH, we speculate conformational changes resulting from the formation of the UreF/UreH/urease complex are necessary to accommodate the UreG dimer. We additionally note that in the GST pull-down experiment demonstrating an interaction between UreG dimer and UreF/UreH/urease complex, UreB (α chain of urease) appears to be substoichiometric in comparison to UreA (β chain of urease) (Figure 7D). This suggests the possibility that some UreB may have dissociated from UreA when interacting with the UreG/UreF/UreH complex and direct interaction occurs between UreG/UreF/UreH and UreA but not UreB. Nevertheless, our results demonstrated that the nickel-charged UreG dimer is able to activate UreF/UreH-bound apo-urease in a GTP-hydrolysis-dependent process (Figure 8A). It is interesting to note that the invariant metal binding motif Cys-Pro-His of UreG is located next to the switch I and II regions (Figure S8). The canonical loaded-spring switch mechanism universal to many GTPases such as Ras and Rho [34] involves conformational changes in switch I and II regions. Given that the Cys-Pro-His motif is in close contact with residues in the switch II region (Figure S8), it can be anticipated that GTP hydrolysis can induce conformational changes at the Cys-Pro-His motif located at the dimeric interface, altering the nickel chelation environment and/or dissociation of UreG into monomer, thus leading to the release of nickel to the urease.

How nickel is transferred to UreG *in vivo* is currently not known. It has been postulated that UreE is the nickel carrier protein and a likely source of nickel in urease maturation [35]–[37]. Structures of UreE with a nickel bound at the interface between two UreE protomers was reported [23],[38] and characterized using X-ray absorption spectroscopy [38]. Moreover, UreE and UreG interaction was detected using yeast-two-hybrid and tandem affinity purification approaches [11]–[13],[39]. Affinity pull-downs showed that *K. aerogenes* UreG and UreE form a complex in the presence of nickel [22]. The strength of the interaction between UreG and UreE was measured using surface plasmon resonance and biolayer interferometry techniques [40]. These observations all support the idea of a nickel transfer between UreE and UreG via protein–protein interaction.

As proposed by Hausinger et al. [26], nickel-carrying UreE may directly transfer nickel to the UreG/UreF/UreH/urease complex (Figure 8B, red arrows). This model is supported by the observation that UreE can interact with UreG/UreF/UreD/urease [22]. It is intriguing that in the crystal structure of UreG/UreF/UreH, the metal binding motif of UreG is buried and there are no obvious channels available in the UreG/UreF/UreH structure for the passage of nickel ions. Given that UreF/UreH can induce large conformational changes in urease [32],[33], conformational changes in the UreG/UreF/UreH/urease complex may occur to allow the transfer of nickel from UreE to the metal binding site of UreG.

Alternatively, the nickel transfer from UreE to UreG occurs before the formation of the UreG/UreF/UreH/urease complex (Figure 8B, blue arrows). Our results suggest that GTP can weaken the interaction between UreG and UreF/UreH (Figure 4A). Since the switch I and II regions of UreG are located near its UreF-interacting residues (Figure 2A), it is conceivable that binding of GTP promotes dissociation of UreG, which then receives a nickel from UreE, perhaps in the form of a (UreE/UreG)_2_ complex as suggested in a modeling attempt by Bellucci et al. [27]. The resulting nickel-charged UreG dimer can then form complex with UreF/UreH and urease. In either case, the preactivation complex, involving nickel-charged UreG, UreF/UreH, and urease, delivers nickel to the urease active site upon stimulation of GTPase activity by bicarbonate (Figure 8A).

As with other GTPase-based molecular switches, it is imperative for UreG to be controlled precisely on the timing of nickel release to function effectively. Previous modeling studies argued that UreF may serve as a GTPase-activating protein (GAP) based on sequence homology [41],[42]. Biagi et al. generated a computational model of the UreE/UreG/UreF/UreH complex [42]. This model depicted UreG in an inverted orientation as compared to what was observed in the crystal structure of the UreG/UreF/UreH complex. The Biagi model suggests that the nucleotide binding site of UreG is in contact with UreF while the metal binding site is away from the UreF interface. However, this is inconsistent with our experimentally determined crystal structure of the UreG/UreF/UreH complex, which showed that the metal binding site is pointing towards the UreF while the nucleotide binding site is far away from the UreF/UreG interface (Figure 1). Moreover, GTPase activity measurements in *K. aerogenes* found no evidence for GAP activity by UreF [16]. Taken together, both pieces of experimental data indicate that it is unlikely for UreF to serve as GAP by supplying an arginine finger for the stimulation of GTP hydrolysis as in the case of Ras [43]. Rather interestingly, we found that it is bicarbonate ion, which is a known cofactor involved in urease maturation [28], that triggers the GTPase activity of UreG, leading to the release of nickel (Figure 6B and 6C) and activation of urease (Figure 7B). Similarly, Boer et al. observed that GTPase activity of UreG in *K. aerogenes* leads to phosphate release in the presence of bicarbonate under *in vitro* conditions [16]. We have shown that the ability to stimulate GTPase activity of UreG is specific for bicarbonate (Figure S6). That other carboxylate-group-containing anions, such as acetate and formate, did not stimulate GTPase activity suggests that it is unlikely for the bicarbonate ion to serve as a surrogate of the carbamylated Lys219 in the urease active site to signal UreG for the release of nickel. Alternatively, it is possible that GTPase activity of UreG is directed for the synthesis of carboxylphosphate as suggested [10] and thereby coupling the carbamylation of active site Lys219 to nickel release from UreG.

Interestingly, it has been reported that in the absence of guanine nucleotide, UreG has a stronger binding affinity to zinc than nickel (K_d_ 0.33 µM versus 10 µM) [21]. Here, our results imply that the nickel affinity of UreG is modulated by guanine-nucleotide binding state. In the absence of free metal ion in the gel filtration buffer, Zn-induced dimer readily dissociated into monomer, while the GTP/nickel-charged UreG dimer remained dimeric (Figure S7). The observation implies that the GTP-bound state of UreG has a stronger binding affinity to nickel than zinc. As shown in the structure of UreG/UreF/UreH, the Cys-Pro-His motif of UreG is located next to the switch I and II regions (Figure S8), and conformational changes upon GTP binding may alter the metal chelating environment to favor nickel binding. It makes biological sense to have a large difference in nickel binding affinity between GDP and GTP bound state of UreG because it provides a mechanism to release the bound nickel upon GTP hydrolysis.

As lucidly argued in a review by Waldron and Robinson, it is the specific protein–protein interactions that drive the delivery of appropriate metal ion to the target metalloproteins [44]. Here, we have shown how urease accessory proteins form a specific complex and interplay with each other to couple GTP hydrolysis to deliver nickel into urease. It is important to recognize that UreG belongs to a growing family of G-proteins regulated by homodimerization [18]. Two other well-characterized nickel-delivering NTPases, namely HypB and CooC1, share strikingly similar properties with UreG. HypB is a close relative of UreG responsible for delivering nickel into hydrogenases. Similar to UreG, it exhibits a varying degree of dimerization in the presence of guanine nucleotides, but achieves complete dimerization in the presence of both GTP and nickel [45]. Crystal structures of HypB in its apo form [46] and GTP/zinc bound form [47] have been reported, showing two invariant cysteine residues located at the dimer interface are responsible for metal chelation. Substitutions that abolished GTP-dependent dimerization of HypB also weaken hydrogenase maturation [46],[48], indicating dimerization is essential to the functioning of HypB. CooC1 is an ATPase responsible for delivery of nickel into carbon monoxide dehydrogenases and a distant relative of UreG in the SIMIBI class NTPases. The presence of nickel, ADP, or ATP induces dimerization of CooC1 [49]. Crystal structure of CooC1 shows that a conserved Cys-X-Cys motif at the dimer interface is responsible for metal chelation [50]. Given the similarity shared among UreG, HypB, and CooC1, we believe that mechanism of nickel delivery described for UreG in this study may represent a general strategy for nickel delivery to other metalloenzymes.

## Materials and Methods

### Protein Expression and Purification

The UreF/UreH complex was purified as described previously [15]. The UreF(R179A/Y183A)/UreH variant (Figure 3B) was purified following the same method as used for the wild-type protein. The expression vector of UreG was constructed by subcloning the *ureG* gene into an in-house pHisSUMO vector, which contains the coding sequence of a HisSUMO tag on a pRSETA vector (Invitrogen). UreG was expressed as a HisSUMO tagged fusion protein using transformed *E. coli*. HisSUMO-UreG expressing bacterial cells were lysed via sonication in buffer A (20 mM Tris/HCl, pH 7.5, 200 mM NaCl, 1 mM TCEP, and 40 mM imidazole) and loaded onto a 5 ml HisTrap column (GE Healthcare) equilibrated with buffer A. After washing with 10 column volumes of buffer A, HisSUMO-UreG was eluted with 300 mM imidazole in buffer A. HisSUMO tag was cleaved using small ubiquitin-like modifier protease SENP1C and separated from UreG by a second pass through the HisTrap column. UreG was further purified by size exclusion chromatography using HiLoad 26/60 Superdex 75 column (GE Healthcare) in buffer A without imidazole. Purified UreG was dialyzed again with 5 mM EDTA to remove any bound metal before further experimentation.

The UreG/UreF/UreH complex was prepared by mixing the UreF/UreH complex with 2-fold molar excess of UreG. The UreG/UreF/UreH complex was then isolated from excess UreG by size exclusion chromatography using HiLoad 26/60 Superdex 200 column (GE Healthcare).

The expression vector for *H. pylori* urease was constructed by subcloning *ureA* and *ureB* into pRSF-duet vector. Urease was expressed using transformed *E. coli*. Bacterial cells containing urease were lysed via sonication in buffer B (20 mM Tris pH 7.5 and 1 mM TCEP) and loaded onto a 5 ml Q Sepharose column (GE Healthcare) equilibrated with buffer B. After extensive washing using buffer B, urease was eluted using a linear 0–500 mM sodium chloride gradient. Pulled fractions were further purified by size exclusion chromatography using HiLoad 26/60 Superdex 200 column (GE Healthcare) in buffer A without imidazole.

For experiments involving the use of nickel-charged UreG dimers (Figures 6, 7, S6, and S7), the UreG dimer was purified by incubating apo-UreG at 3 mg/ml on ice with 1 mM GTP, 2 mM magnesium sulfate, and 0.5 mM nickel sulfate for 15 min. The nickel-charged UreG dimer formed was then separated from excess GTP and nickel using a Sephadex G25 desalting column.

### Crystallization and Structural Determination of the UreG/UreF/UreH Complex

The UreG/UreF/UreH complex was concentrated to 20 mg/ml for crystallization. Just before crystallization, 5 mM GDP, 10 mM magnesium sulfate, and 5 mM aluminum fluoride was added to the UreG/UreF/UreH complex. Crystals of the UreG/UreF/UreH complex was grown using sitting drop vapor diffusion method in 1.5 M ammonium sulfate and 100 mM sodium acetate pH 5.0 at 16°C. Crystals were harvested after 2 wk and cryoprotected by soaking in mother liquor containing 20% glycerol before flash freezing with liquid nitrogen for data collection. X-ray diffraction data were collected on beamline I-04 at a wavelength of 0.9795 Å at the Diamond Light Source (Oxfordshire, UK). Diffraction data were integrated and scaled using XDS [51]. Initial phases were obtained using molecular replacement with PHASER [52]. Structures of the UreF/UreH complex (PDB code: 3SF5) and HypB (PDB code: 2HF9) devoid of the GTPγS ligand were used as molecular replacement search models. The resulting model was further improved by iterative rounds of refinement using PHENIX.REFINE [53] with noncrystallographic symmetry (NCS) restrains and manual model building with COOT [54]. After initial rounds of refinement, clear positive electron density was identified in the Fo-Fc map in the guanine nucleotide binding pocket of UreG, in which GDP was modeled (Figure S9A). Translation-libration-screw (TLS) refinement procedure was applied in the final rounds of refinement. 2Fo-Fc map shows that clear electron density for the metal binding motif of UreG was observed (Figure S9B). The Molprobity [55] validated final model has 97.84% and 2.16% in Ramachandran favored and allowed regions, with no outlier residues.

### GST Pull-Down Assay

To detect interaction of the UreF/UreH complex with UreG (Figure 3C), bacterial cells expressing UreG and those expressing HisGST-UreF/UreH or HisGST-UreF(R179A/Y183D)/UreH complexes were mixed and sonicated together. Cleared cell lysate was loaded onto GST SpinTrap column (GE Healthcare) and incubated for 30 min at 4°C. The column was then washed 5 times with 500 µl of buffer C (20 mM Tris pH 7.5, 200 mM sodium chloride, 1 mM TCEP), followed by elution using buffer C with 10 mM glutathione. Eluted protein was analyzed using 15% SDS-PAGE.

To study the interaction between UreF/UreH and UreG in the presence of guanine nucleotide and/or nickel/zinc ions (Figures 4A and S4), purified GST-UreF/UreH and excess UreG was mixed and loaded onto GST SpinTrap column (GE Healthcare). After incubating for 30 min at 4°C and washing away unbound proteins, the immobilized GST-UreG/UreF/UreH complex was incubated with 1 mM GTP or GDP, 2 mM magnesium sulfate, and/or 0.5 mM nickel sulfate for 30 min at 4°C. Column was then washed 5 times with 500 µl of buffer C, followed by elution using buffer C with 10 mM glutathione. The wash fraction and eluted fraction was collected and analyzed using 15% SDS-PAGE.

To study the interaction between nickel-charged UreG dimer, UreF/UreH, and UreF/UreH/urease (Figure 7D), purified GST-UreF/UreH or GST-UreF/UreH mixed with overexpressed urease lysate was loaded onto 5 ml GSTrap column (GE Healthcare). After extensive washing with buffer C, apo-UreG or nickel-charged UreG dimer was injected into the column and incubated for 15 min. Column was then washed again with buffer C to remove unbound UreG and finally eluted using buffer C with 10 mM glutathione.

### Size Exclusion Chromatography/Static Light Scattering (SEC/SLS)

For the study of dimerization-deficient UreF variant (Figure 3), 100 µl of 50 µM purified UreF/UreH complex or its mutants were injected into a Superdex 200 analytical gel filtration column pre-equilibrated with phosphate-buffered saline. For the study of nickel/guanine nucleotide–dependent dissociation of UreG from UreF/UreH (Figure 4B) and dimerization of UreG (Figure 5A and 5B), 100 µl of 40 µM UreG or UreG/UreF/UreH complex pre-incubated with different combinations of 1 mM GTP/GDP, 2 mM magnesium sulfate, and 0.5 mM nickel sulfate was injected into the same column. For the study of bicarbonate-induced change in oligomerization state of nickel-charged UreG (Figure 6A), 75 µM of nickel-charged UreG with or without prior incubation of 100 mM bicarbonate was injected into the same column.

For testing the metal specificity of UreG dimerization (Figure S7), experimental conditions were chosen to closely match that used by Zambelli et al. [21] to make our results comparable. We pre-incubated 100 µM of UreG with 1 mM magnesium sulfate, 100 µM nickel sulfate, or zinc sulfate in the presence/absence of 0.5 mM GDP or GTP. The protein samples were injected into a Superdex 200 analytical gel filtration column pre-equilibrated the gel filtration buffer containing 20 mM Tris pH 8.0 and 150 mM NaCl. We included 10 µM of nickel or zinc in the gel filtration buffer in selected injections.

In all cases, protein eluted off the gel filtration column was fed into a downstream miniDawn light scattering detector and an Optilab DSP refractometer (Wyatt Technologies). Data were analyzed using ASTRA software provided by the manufacturer (Wyatt Technologies), and the molecular weights measured along with the fitting errors are reported.

### Atomic Absorption Spectroscopy (AAS)

UreG injected into the Superdex 200 analytical gel filtration/static light scattering system was eluted into a 4 ml fraction. Nitric acid was added to the eluted UreG to a final concentration of 1%. Nickel concentration was measured using Hitachi Z-2700 polarized Zeeman atomic absorption spectrometer with graphite furnace. Data were analyzed using software provided by the manufacturer. Nickel concentration was determined by comparing measurements against a standard curve of nickel known concentration (Figures 5C and 6C).

### Malachite Green Assay

We incubated 200 µl of 75 µM of nickel-charged UreG in the absence or presence of 100 mM sodium bicarbonate for 3 h at 37°C. Phosphate released from UreG GTPase activity was measured using a colorimetric assay method based on malachite green [56]. Measurements were compared against a standard curve prepared using known amounts of phosphate to determine the phosphate concentration. Phosphate released by UreG dimer was derived from the difference between the amount of phosphate in solution before and after the incubation period (Figures 6B, S5, and S6).

### *In Vivo* Urease Activation Assay

For the study of dimerization-deficient UreF variant, a cell lysate-based urease activity assay was used (Figure 3D). pHpA2H vector was constructed by cloning the entire urease operon into a pRSETA vector. pHpA2H mutant plasmids were constructed using Quikchange site-directed mutagenesis protocol (Strategene). pHpA2H plasmids carrying mutations on either *ureF* gene were transformed into *E. coli* cells. Urease activity assay was performed as described [15].

### *In Vitro* Urease Activation Assay

For the study of investigating the ability of nickel-charged UreG in urease activation, an *in vitro* urease activity assay using purified proteins was used (Figure 7). The standard buffer for *in vitro* activation of urease consists of 20 mM HEPES pH 7.5, 150 mM sodium chloride, 1 mM TCEP, and 100 mM sodium bicarbonate. To test the activation of urease, 20 µM of each of purified urease and UreF/UreH complex was incubated with 20 µM nickel-charged UreG dimers for 2 h at 37°C. Urease activity was then determined by incubating the enzyme with 25 mM urea for 30 min at 37°C and then measuring the ammonia released using a phenol/hypochlorite reaction as described [57].

## Supporting Information

Figure S1 Superposition of UreF and UreH chains observed in crystal structures of UreF/UreH and UreG/UreF/UreH. UreF and UreH chains found in crystal structures of UreF/UreH (white, PDB: 3SF5) and UreG/UreF/UreH (red, PDB: 4HI0) are superposed. Cα root mean square deviation the UreF and UreH chains is 0.742 Å, and there are no major conformational changes on UreF and UreH upon complex formation with UreG.
(TIF)

Figure S2 Topology of UreG and its guanine nucleotide binding pocket. (A) Topology diagram of UreG. Loop regions constituting the P-loop (G1), switch I (G2), switch II (G3), G4, G5, and metal binding motif are colored yellow, blue, cyan, purple, orange, and green, respectively. Note that UreG has a seven-stranded β sheet, which is characteristic of all SIMIBI class GTPases. (B) A close-up view of the guanine nucleotide binding pocket. GDP is shown in stick representation. G1–G5 motifs are colored as in (A). Residues involved in binding guanine nucleotide are shown in stick representation. α and β phosphate are wrapped around by the main chain amide groups of the P-loop (G1), typical of most NTPases. Most other SIMIBI and TRAFAC class GTPases have a strictly conserved switch II (G3) motif of DXXG, in which the aspartate side chain is responsible for a water-mediated chelation of magnesium ion. The G3E family, to which UreG belongs, uses an alternative switch II motif of EXXG with the glutamate side chain directly participating in chelating magnesium ion. In the case of UreG, switch II consists of a totally invariant ^98^ESGGDNL^104^ motif. UreG recognizes guanine nucleotide using the canonical NKXD motif (G4), in which Asp-148 forms a bifurcated hydrogen bond with N1 and N2 atoms of guanine ring. O6 oxygen atom of the guanine ring is stabilized by Ile-178 and Asn-145, which is part of the G5 motif. Aliphatic regions of Arg-179 and Lys-146 make extensive hydrophobic interactions with the guanine ring.
(TIF)

Figure S3 Sequence alignment of UreG with secondary structural elements labeled: *Helicobacter pylori* (HELPY); *Klebsiella aerogenes* (ENTAE); *Bacillus pasteurii* (BACPA); *Bacillus halodurans* (BACHD); *Clostridium phytofermentans* (CLOPH); *Synechococcus sp.* (strain JA-3-3Ab) (SYNJA); *Silicibacter pomeroyi* (SILPO); *Opitutus terrae* (OPITP); *Arcobacter butzleri* (ARCB4). Residues were colored in different shades of black according to the degree of conservation, with the darkest color representing the most conserved residues. GTPase structural motifs (G1–G5) and metal binding motif (Metal) are labeled and colored as in Figure 2A. Residues involved in interacting with UreF are indicated as red triangles.
(TIF)

Figure S4 GTP and Zinc induces dissociation of UreG from the UreF/UreH complex. The GST-UreF/UreH/UreG complex was first immobilized on GST Spintrap columns. After washing with 0.5 mM zinc and/or 1 mM GDP/GTP, proteins remained on the column were eluted with glutathione. The wash (W) and eluted (E) fractions were analyzed using SDS-PAGE. UreG completely dissociated from UreF/UreH complex in the presence of zinc and GTP.
(TIF)

Figure S5 GTP hydrolysis in the presence or absence of UreG. We incubated 75 µM of each of GTP and nickel (left) or 75 µM of nickel-charged UreG dimer (right) was incubated in the absence or presence of 100 mM sodium bicarbonate for 3 h at 37°C. Phosphate released from UreG GTPase activity was measured using a colorimetric assay method based on malachite green [56]. In contrast to nickel-charged UreG dimer, only a negligible amount of GTP was hydrolyzed without UreG.
(TIF)

Figure S6 Stimulation of UreG GTPase activity is specific to bicarbonate. To test the specificity of bicarbonate stimulation of UreG GTPase activity, we incubated 75 µM of nickel-charged UreG dimer in the presence of 100 mM bicarbonate, acetate formate, or sulfate for 3 h at 37°C. Significant GTP hydrolysis only occurred in the presence of bicarbonate.
(TIF)

Figure S7 Nickel induced GTP-dependent dimerization of UreG. We pre-incubated 100 µM UreG with 100 µM nickel sulfate (left panel) or zinc sulfate (right panel) in the presence/absence of 0.5 mM guanine nucleotides. The protein samples were injected to Superdex S200 analytical gel filtration column. In (A), UreG was eluted using gel filtration buffer containing 20 mM Tris pH 8.0 and 150 mM NaCl (metal free gel filtration buffer; black lines). In (B), UreG was eluted using gel filtration buffer with additional 10 µM of nickel or zinc (nickel/zinc gel filtration buffer; red lines). Molecular weights measured by SEC/SLS were indicated.
(TIF)

Figure S8 UreG metal binding motif is in close proximity to GTPase switch I and switch II motifs. GDP ligand, P-loop (yellow), Switch I (blue), switch II (cyan), and metal binding motifs (green) of UreG are shown as sticks and indicated. Hydrogen bond formed between His66 and Asn103 is indicated by yellow dash line.
(TIF)

Figure S9 Electron density maps of selected regions of UreG structure. (A) Fo-Fc electron density map (contoured at 3.0 σ) before the GDP ligand (yellow) was modeled. Two UreG protomers are colored in white and grey, respectively. (B) Stereoview of 2Fo-Fc electron density map (contoured at 1.2 σ) of the invariant Cys-Pro-His metal binding motif of UreG at its dimeric interface. Residue Cys-66 and His-68 of the invariant metal binding Cys-Pro-His motif are highlighted in green.
(TIF)

Table S1 Data collection and refinement statistics.
(DOC)

Table S2 Summary of hydrogen bonds and salt bridges between UreG and UreF.
(DOC)

## Abbreviations

AAS: atomic absorption spectroscopy
EDTA: ethylenediaminetetraacetic acid
GAP: GTPase-activating protein
GDP: guanosine diphosphate
GST: glutathione S-transferase
GTP: guanosine triphosphate
GTPγS: guanosine 5′-O-[gamma-thio]triphosphate
HisGST: hexahistidine tagged glutathione S-transferase
HisSUMO: hexahistidine tagged small ubiquitin-like modifier protein
PDB: Protein Data Bank
SAXS: small angle x-ray scattering
SEC/SLS: size exclusion chromatography/static light scattering
SIMIBI: signal recognition particle, MinD, BioD
SDS-PAGE: sodium dodecyl sulfate polyacrylamide gel electrophoresis
TCEP: tris(2-carboxyethyl)phosphine
Tris: tris(hydroxymethyl)aminomethane
