# Supplementary figures and images for: Structure of UreG/UreF/UreH Complex Reveals How Urease Accessory Proteins Facilitate Maturation of Helicobacter pylori Urease
Source: PLoS Biol. 2013 Oct 8;11(10):e1001678. doi: 10.1371/journal.pbio.1001678 (PMC3792862; doi:10.1371/journal.pbio.1001678)

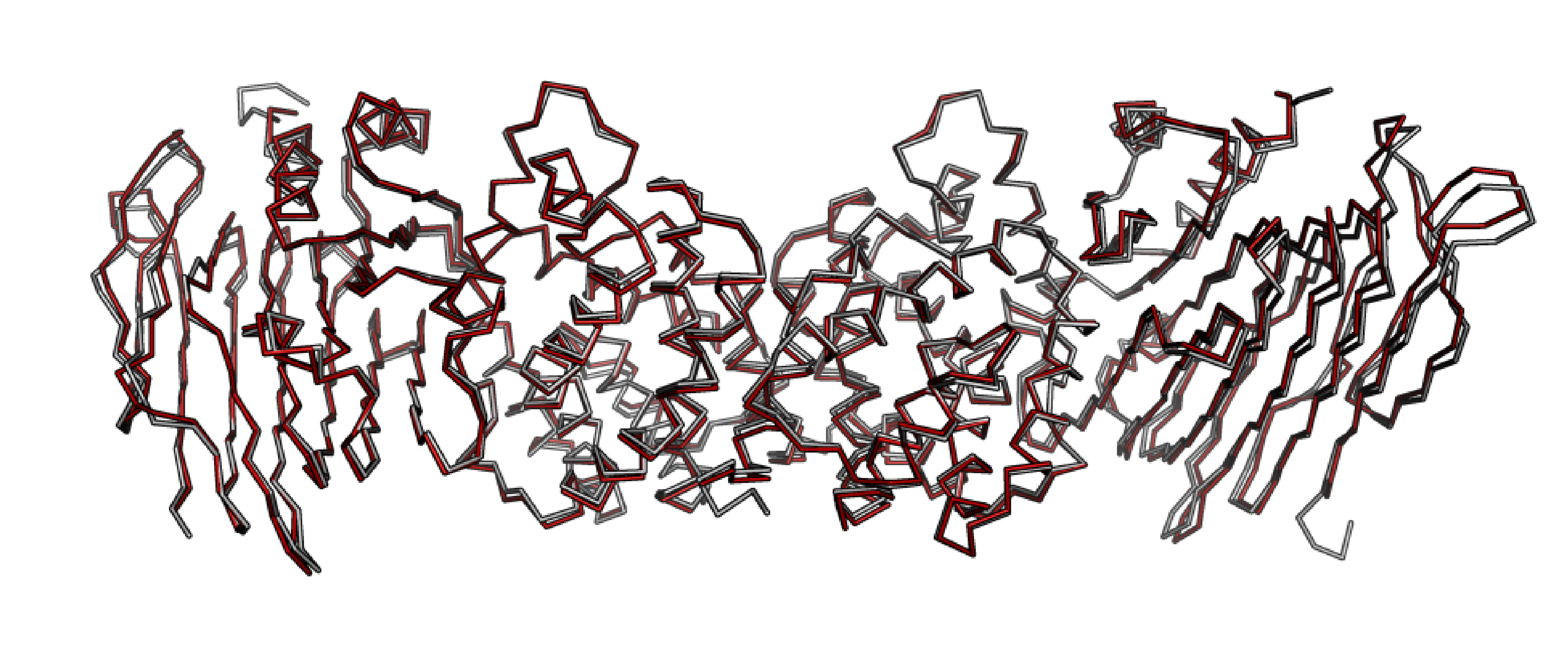

Supplement: Figure S1 — Superposition of UreF and UreH chains observed in crystal structures of UreF/UreH and UreG/UreF/UreH. UreF and UreH chains found in crystal structures of UreF/UreH (white, PDB: 3SF5) and UreG/UreF/UreH (red, PDB: 4HI0) are superposed. Cα root mean square deviation the UreF and UreH chains is 0.742 Å, and there are no major conformational changes on UreF and UreH upon complex formation with UreG. (TIF) [file pbio.1001678.s001.tif]

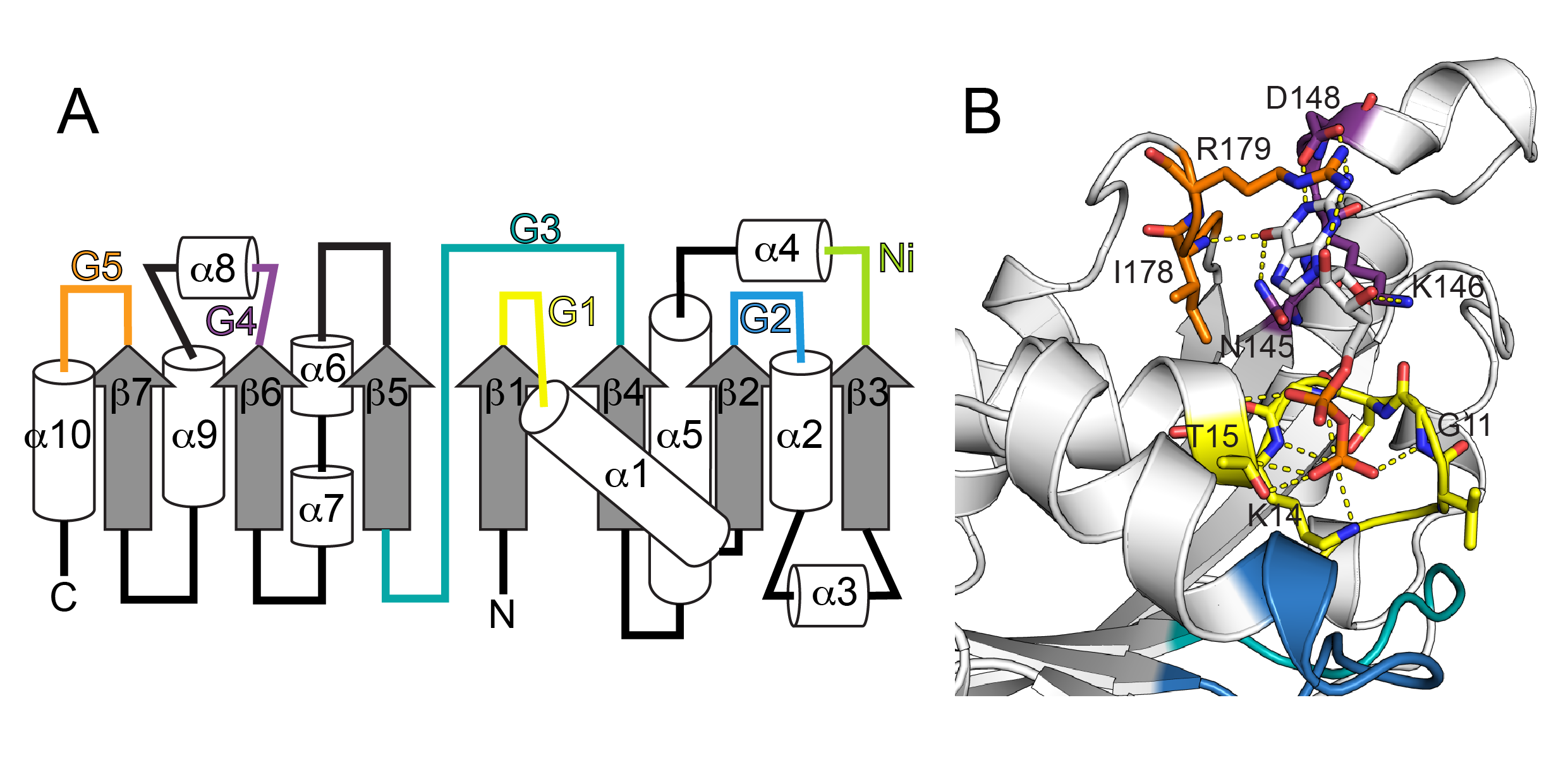

Supplement: Figure S2 — Topology of UreG and its guanine nucleotide binding pocket. (A) Topology diagram of UreG. Loop regions constituting the P-loop (G1), switch I (G2), switch II (G3), G4, G5, and metal binding motif are colored yellow, blue, cyan, purple, orange, and green, respectively. Note that UreG has a seven-stranded β sheet, which is characteristic of all SIMIBI class GTPases. (B) A close-up view of the guanine nucleotide binding pocket. GDP is shown in stick representation. G1–G5 motifs are colored as in (A). Residues involved in binding guanine nucleotide are shown in stick representation. α and β phosphate are wrapped around by the main chain amide groups of the P-loop (G1), typical of most NTPases. Most other SIMIBI and TRAFAC class GTPases have a strictly conserved switch II (G3) motif of DXXG, in which the aspartate side chain is responsible for a water-mediated chelation of magnesium ion. The G3E family, to which UreG belongs, uses an alternative switch II motif of EXXG with the glutamate side chain directly participating in chelating magnesium ion. In the case of UreG, switch II consists of a totally invariant 98ESGGDNL104 motif. UreG recognizes guanine nucleotide using the canonical NKXD motif (G4), in which Asp-148 forms a bifurcated hydrogen bond with N1 and N2 atoms of guanine ring. O6 oxygen atom of the guanine ring is stabilized by Ile-178 and Asn-145, which is part of the G5 motif. Aliphatic regions of Arg-179 and Lys-146 make extensive hydrophobic interactions with the guanine ring. (TIF) [file pbio.1001678.s002.tif]

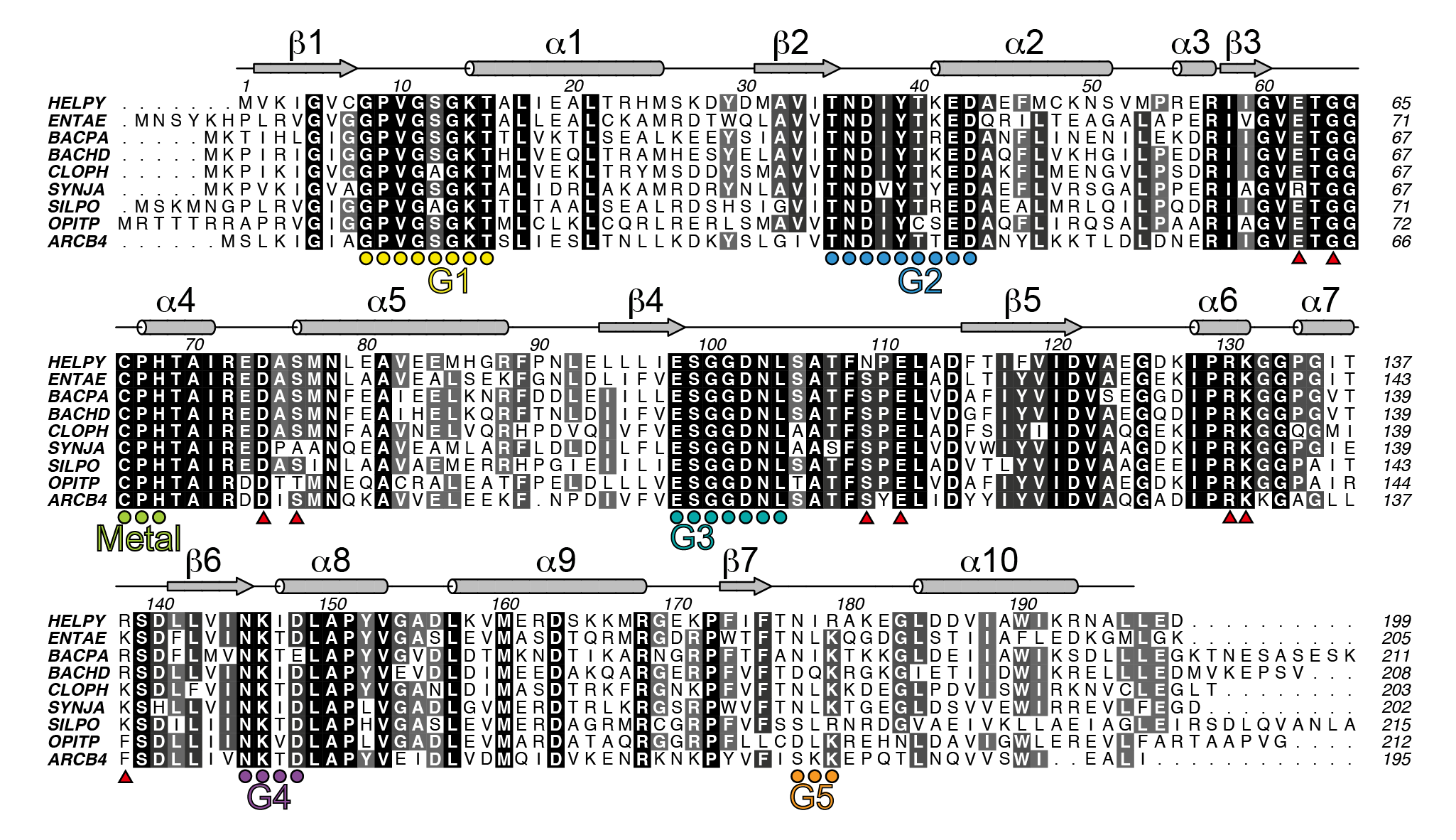

Supplement: Figure S3 — Sequence alignment of UreG with secondary structural elements labeled: Helicobacter pylori (HELPY); Klebsiella aerogenes (ENTAE); Bacillus pasteurii (BACPA); Bacillus halodurans (BACHD); Clostridium phytofermentans (CLOPH); Synechococcus sp. (strain JA-3-3Ab) (SYNJA); Silicibacter pomeroyi (SILPO); Opitutus terrae (OPITP); Arcobacter butzleri (ARCB4). Residues were colored in different shades of black according to the degree of conservation, with the darkest color representing the most conserved residues. GTPase structural motifs (G1–G5) and metal binding motif (Metal) are labeled and colored as in Figure 2A. Residues involved in interacting with UreF are indicated as red triangles. (TIF) [file pbio.1001678.s003.tif]

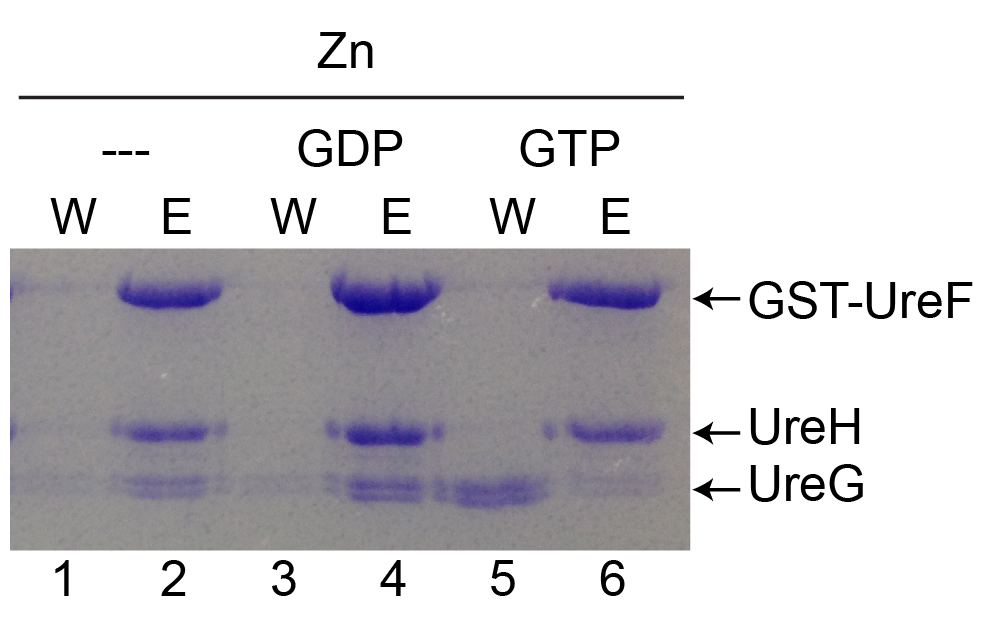

Supplement: Figure S4 — GTP and Zinc induces dissociation of UreG from the UreF/UreH complex. The GST-UreF/UreH/UreG complex was first immobilized on GST Spintrap columns. After washing with 0.5 mM zinc and/or 1 mM GDP/GTP, proteins remained on the column were eluted with glutathione. The wash (W) and eluted (E) fractions were analyzed using SDS-PAGE. UreG completely dissociated from UreF/UreH complex in the presence of zinc and GTP. (TIF) [file pbio.1001678.s004.tif]

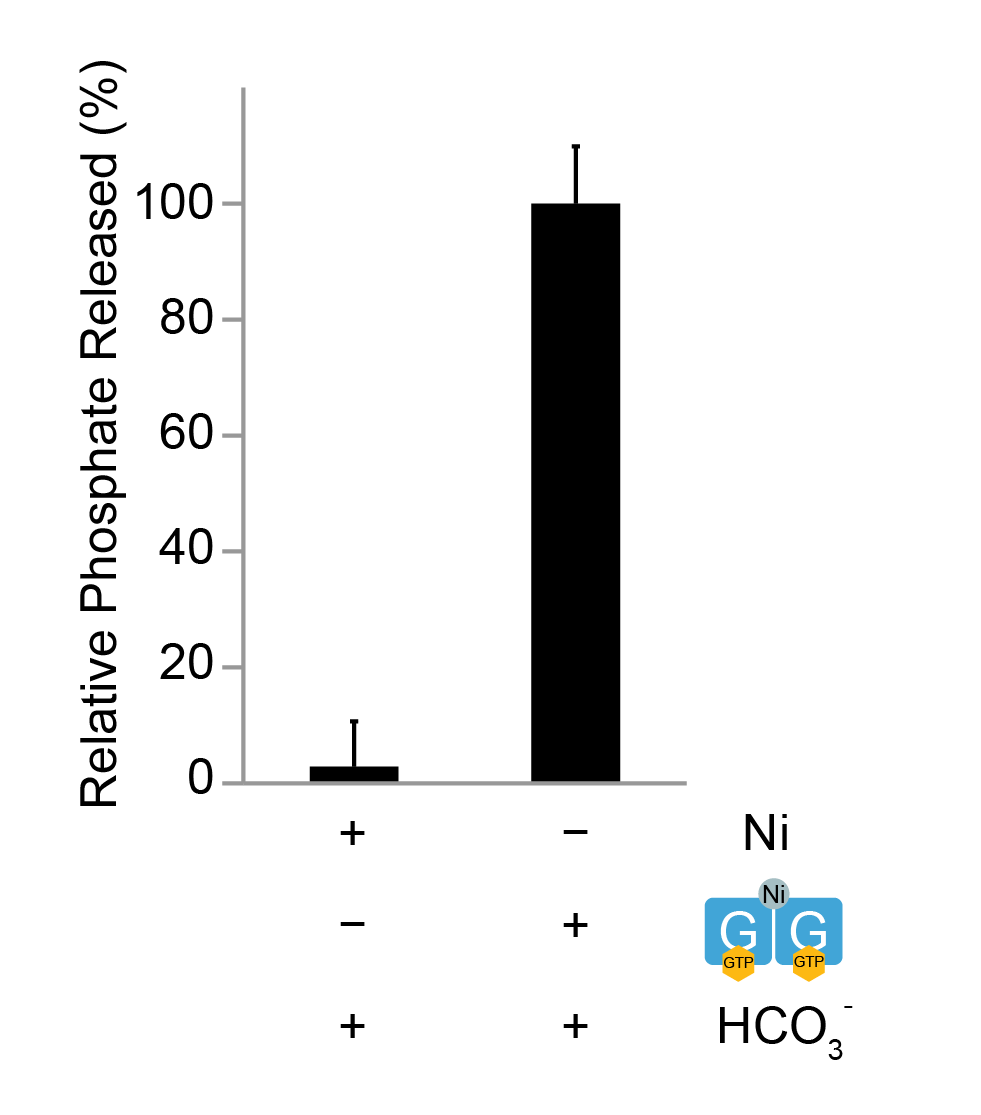

Supplement: Figure S5 — GTP hydrolysis in the presence or absence of UreG. We incubated 75 µM of each of GTP and nickel (left) or 75 µM of nickel-charged UreG dimer (right) was incubated in the absence or presence of 100 mM sodium bicarbonate for 3 h at 37°C. Phosphate released from UreG GTPase activity was measured using a colorimetric assay method based on malachite green [56]. In contrast to nickel-charged UreG dimer, only a negligible amount of GTP was hydrolyzed without UreG. (TIF) [file pbio.1001678.s005.tif]

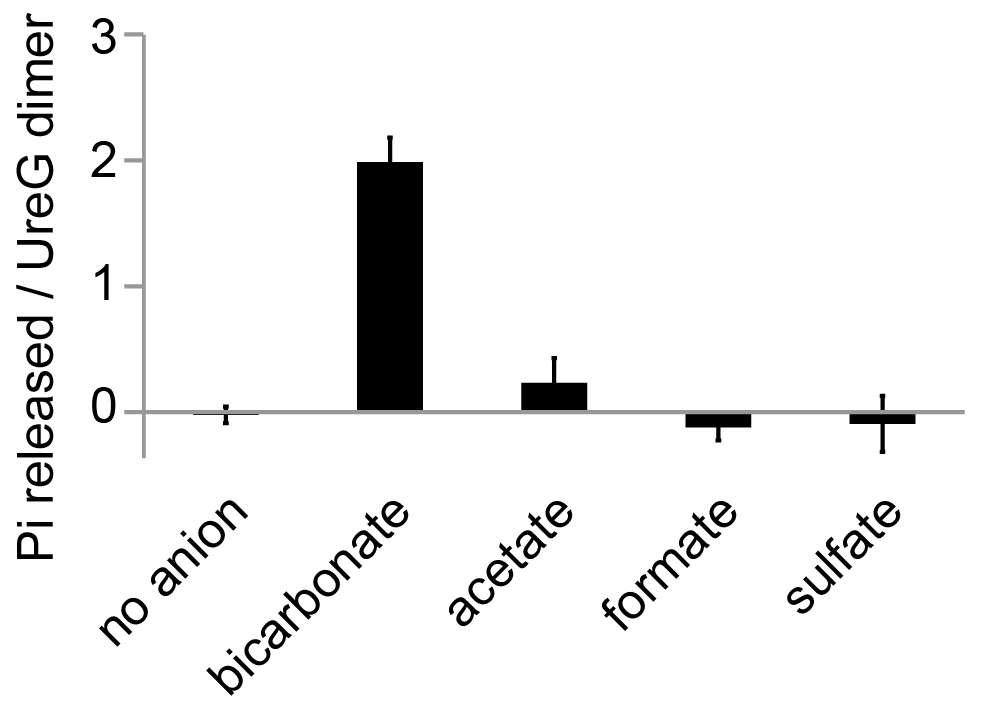

Supplement: Figure S6 — Stimulation of UreG GTPase activity is specific to bicarbonate. To test the specificity of bicarbonate stimulation of UreG GTPase activity, we incubated 75 µM of nickel-charged UreG dimer in the presence of 100 mM bicarbonate, acetate formate, or sulfate for 3 h at 37°C. Significant GTP hydrolysis only occurred in the presence of bicarbonate. (TIF) [file pbio.1001678.s006.tif]

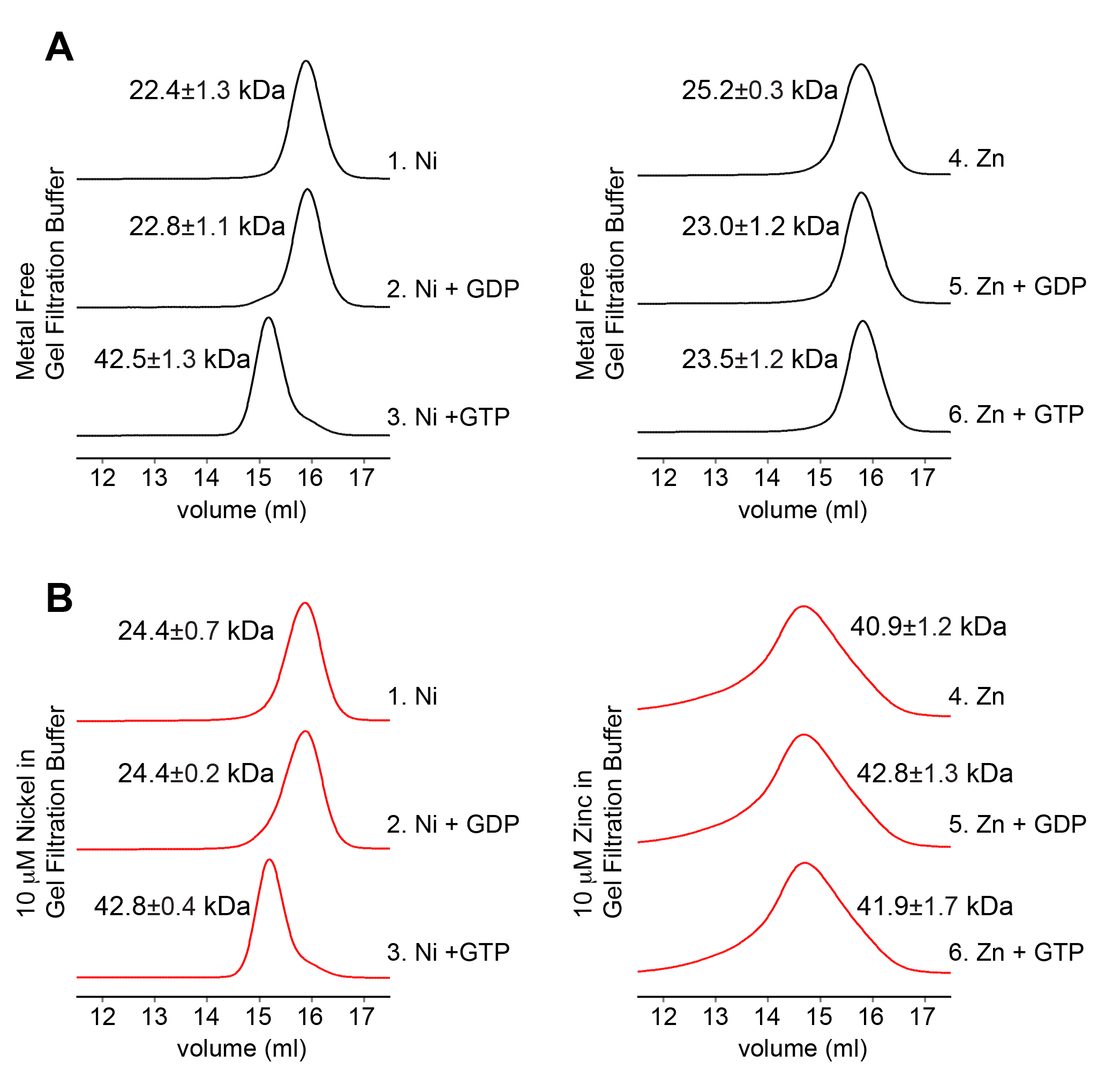

Supplement: Figure S7 — Nickel induced GTP-dependent dimerization of UreG. We pre-incubated 100 µM UreG with 100 µM nickel sulfate (left panel) or zinc sulfate (right panel) in the presence/absence of 0.5 mM guanine nucleotides. The protein samples were injected to Superdex S200 analytical gel filtration column. In (A), UreG was eluted using gel filtration buffer containing 20 mM Tris pH 8.0 and 150 mM NaCl (metal free gel filtration buffer; black lines). In (B), UreG was eluted using gel filtration buffer with additional 10 µM of nickel or zinc (nickel/zinc gel filtration buffer; red lines). Molecular weights measured by SEC/SLS were indicated. (TIF) [file pbio.1001678.s007.tif]

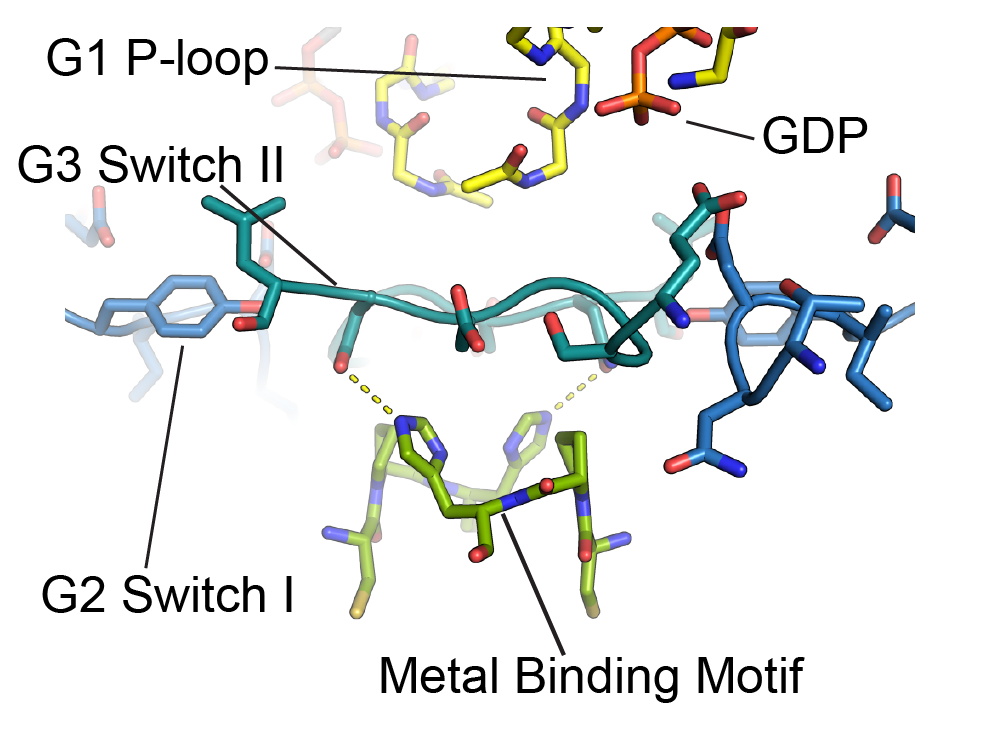

Supplement: Figure S8 — UreG metal binding motif is in close proximity to GTPase switch I and switch II motifs. GDP ligand, P-loop (yellow), Switch I (blue), switch II (cyan), and metal binding motifs (green) of UreG are shown as sticks and indicated. Hydrogen bond formed between His66 and Asn103 is indicated by yellow dash line. (TIF) [file pbio.1001678.s008.tif]

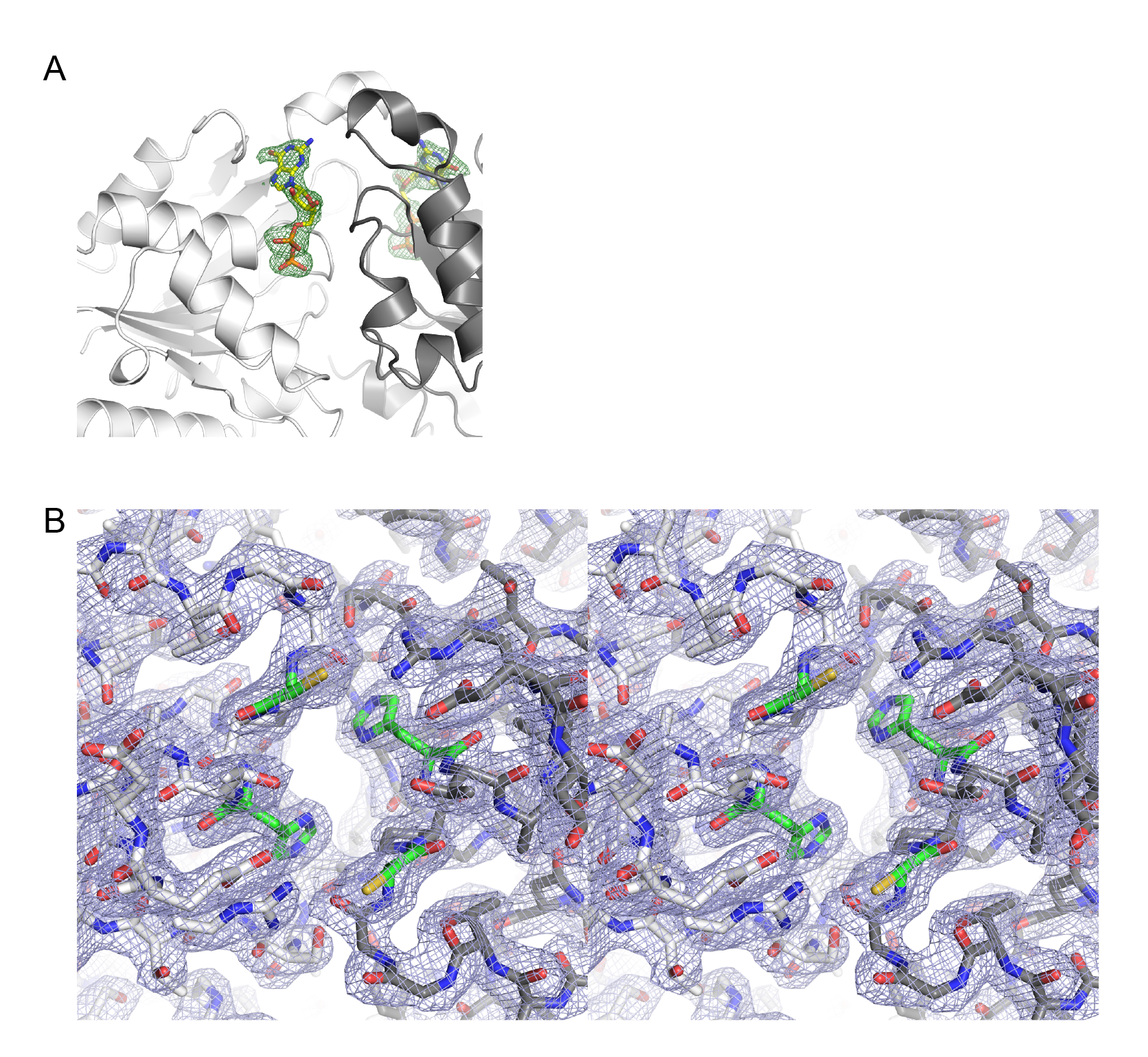

Supplement: Figure S9 — Electron density maps of selected regions of UreG structure. (A) Fo-Fc electron density map (contoured at 3.0 σ) before the GDP ligand (yellow) was modeled. Two UreG protomers are colored in white and grey, respectively. (B) Stereoview of 2Fo-Fc electron density map (contoured at 1.2 σ) of the invariant Cys-Pro-His metal binding motif of UreG at its dimeric interface. Residue Cys-66 and His-68 of the invariant metal binding Cys-Pro-His motif are highlighted in green. (TIF) [file pbio.1001678.s009.tif]
