# Supplementary material for: Structure of UreG/UreF/UreH Complex Reveals How Urease Accessory Proteins Facilitate Maturation of Helicobacter pylori Urease
Source: PLoS Biol. 2013 Oct 8;11(10):e1001678. doi: 10.1371/journal.pbio.1001678 (PMC3792862; doi:10.1371/journal.pbio.1001678)
Supplement: Table S1 — Data collection and refinement statistics. (DOC) [file pbio.1001678.s010.doc]

**Table S1**

**Data Collection and Refinement Statistics.**

|  | PDB Code: 4HI0 |
| --- | --- |
| **Data collection** |  |
| Space group | P2_1_2_1_2_1_ |
| Cell dimensions |  |
| *a*, *b*, *c* (Å) | 76.12, 96.43, 237.45 |
| α, β, γ (°) | 90, 90, 90 |
| Resolution (Å) | 30.00-2.35 (2.49-2.35) |
| *R*_merge_ | 0.065 (0.365) |
| *I* / σ*I* | 15.93 (3.86) |
| Completeness (%) | 99.5 (99.3) |
| Redundancy | 3.6 (3.7) |
|  |  |
| **Refinement** |  |
| Resolution (Å) | 30.00-2.35 |
| No. reflections | 69047 |
| *R*_work_ / *R*_free_ | 0.192 / 0.242 |
| No. atoms |  |
| Protein | 11005 |
| Water | 303 |
| *B*-factors |  |
| Protein | 50.56 |
| Water | 39.39 |
| R.m.s. deviations |  |
| Bond lengths (Å) | 0.008 |
| Bond angles (°) | 1.187 |

*Values in parentheses are for highest-resolution shell.
