# Supplementary material for: Structure of UreG/UreF/UreH Complex Reveals How Urease Accessory Proteins Facilitate Maturation of Helicobacter pylori Urease
Source: PLoS Biol. 2013 Oct 8;11(10):e1001678. doi: 10.1371/journal.pbio.1001678 (PMC3792862; doi:10.1371/journal.pbio.1001678)
Supplement: Table S2 — Summary of hydrogen bonds and salt bridges between UreG and UreF. (DOC) [file pbio.1001678.s011.doc]

**Table S2**

**Summary of hydrogen bonds and salt bridges between UreG and UreF.**

| UreG (chain E) | UreF(chain A) | Distance (Å) |
| --- | --- | --- |
| Asn109 [ND2] | Leu199 [O] | 2.7 |
| Glu111 [OE2] | Ser200 [OG] | 3.1 |
| Arg130 [O] | Tyr248 [OH] | 2.6 |
| Lys131 [O] | Ser249 [OG] | 3.1 |
| Arg138 [NH2] | Val197 [O] | 3.1 |
| UreG (chain E) | UreF(chain C) | Distance (Å) |
| Glu62 [OE2] | Arg250 [NH2] | 3.0 |
| Gly64 [N] | Leu251 [O] | 2.9 |
| Asp74 [O] | Tyr48 [OH] | 2.8 |
| Asp74 [OD2] | Gly46 [N] | 2.9 |
| Ser76 [OG] | Pro44 [O] | 2.8 |
